# Normative model detects abnormal functional connectivity in psychiatric disorders

**DOI:** 10.3389/fpsyt.2023.1068397

**Published:** 2023-02-15

**Authors:** Duarte Oliveira-Saraiva, Hugo Alexandre Ferreira

**Affiliations:** Institute of Biophysics and Biomedical Engineering, Faculty of Sciences of the University of Lisbon, Lisbon, Portugal

**Keywords:** normative model, functional brain network, deep learning, psychiatric disorders, functional connectivity

## Abstract

**Introduction:**

The diagnosis of psychiatric disorders is mostly based on the clinical evaluation of the patient's signs and symptoms. Deep learning binary-based classification models have been developed to improve the diagnosis but have not yet reached clinical practice, in part due to the heterogeneity of such disorders. Here, we propose a normative model based on autoencoders.

**Methods:**

We trained our autoencoder on resting-state functional magnetic resonance imaging (rs-fMRI) data from healthy controls. The model was then tested on schizophrenia (SCZ), bipolar disorder (BD), and attention-deficit hyperactivity disorder (ADHD) patients to estimate how each patient deviated from the norm and associate it with abnormal functional brain networks' (FBNs) connectivity. Rs-fMRI data processing was conducted within the FMRIB Software Library (FSL), which included independent component analysis and dual regression. Pearson's correlation coefficients between the extracted blood oxygen level-dependent (BOLD) time series of all FBNs were calculated, and a correlation matrix was generated for each subject.

**Results and discussion:**

We found that the functional connectivity related to the basal ganglia network seems to play an important role in the neuropathology of BD and SCZ, whereas in ADHD, its role is less evident. Moreover, the abnormal connectivity between the basal ganglia network and the language network is more specific to BD. The connectivity between the higher visual network and the right executive control and the connectivity between the anterior salience network and the precuneus networks are the most relevant in SCZ and ADHD, respectively. The results demonstrate that the proposed model could identify functional connectivity patterns that characterize different psychiatric disorders, in agreement with the literature. The abnormal connectivity patterns from the two independent SCZ groups of patients were similar, demonstrating that the presented normative model was also generalizable. However, the group-level differences did not withstand individual-level analysis implying that psychiatric disorders are highly heterogeneous. These findings suggest that a precision-based medical approach, focusing on each patient's specific functional network changes may be more beneficial than the traditional group-based diagnostic classification.

## 1. Introduction

The diagnosis of psychiatric disorders is still exclusively dependent on the analysis of the signs and symptoms of the patient, and it is mostly based on the Diagnostic and Statistical Manual of Mental Disorders 5 (DSM-5) (1), as no biomarkers have proven useful in clinical practice (2). The DSM-5 authors recognized that psychiatric disorders do not always fit completely within the boundaries of a single disorder, with several symptom domains involving multiple diagnostic categories. Thus, a high rate of patients are misdiagnosed (3–5). In fact, Ayano et al. found that three out of four schizoaffective patients, one out of two major depressive disorder patients, one out of four schizophrenia (SCZ) patients, and one out of five bipolar disorder (BD) patients are misdiagnosed. Besides, patients with BD are more likely to be misdiagnosed as having SCZ, whereas SCZ patients are more likely to be misdiagnosed as having BD (4). In addition, it is estimated that 70–85% of individuals diagnosed with attention-deficit hyperactivity disorder (ADHD) meet criteria for at least one other psychiatric disorder (5). As a consequence, therapeutic approaches do not always succeed, affecting patients' quality of life. In order to tackle the challenge of diagnosis, deep learning (DL) strategies have been studied (6). Although psychiatric disorders develop over a continuous spectrum and multiple disorders can coexist in a patient, most research studies make use of binary classification (6, 7). In addition, some DL algorithms have been criticized due to the difficulty of explaining how they work. Even when great accuracy is attained, it is difficult to pinpoint the traits that contribute the most to a particular diagnosis. As a result, nothing is learned about the disorders' underlying mechanisms (8). Consequently, the translation of those algorithms to clinical practice is compromised since they are more concerned with identifying the group to which the patient belongs even when the boundaries of psychiatric disorders are undefined. Additionally, they give no information about the abnormalities of the particular subject, which is essential for the application of the right therapies. To face the continuous spectrum of such disorders, alternative approaches must be applied.

The normative model is an emerging approach that describes and quantifies how individuals deviate from an expected pattern learned from a population (9). As such, an algorithm is used to learn patterns from healthy individuals resulting in a model of “normality” that then is tested on healthy subjects and patients for anomaly detection. Subjects with abnormal brain patterns concerning this normal range may be identified as outliers (8). The main benefits of this approach are that it allows for the identification of pathological patterns underlying a variety of disorders and moreover it has the potential to deal with the heterogeneity of psychiatric disorders (8, 10). Several algorithms have been used to generate normative models, such as Gaussian process regression, generative adversarial networks, and autoencoders [for more information see (10, 11)]. Most studies that applied the normative model approach to neuroimaging used structural magnetic resonance imaging (MRI) data (10). Additionally, only one of the three studies that used functional MRI (fMRI) (12–14), focused on functional connectivity metrics (14). Considering that several psychiatric disorders have been associated with the hypothesis of abnormal functional connectivity, there is much to be explored about combining functional brain data with the normative model approach.

Taking into account the available public datasets, we decided to demonstrate the usage of such normative model with three related psychiatric disorders: ADHD; BD; SCZ.

ADHD is considered a neurodevelopment disorder and is characterized by impairing levels of inattention, disorganization, hyperactivity, and impulsivity (1). It impairs several aspects of life, affecting social relationships, as well as academic and work performance (15). In addition, mild delays in language, motor, or social development often occur (1).

Castellanos and Aoki (16) did a review study on functional connectivity in ADHD. From their analysis, they highlighted that ADHD may be characterized by a decreased synchrony between the anterior and posterior nodes of the default mode network and by the abnormal interplay between several functional brain networks (FBNs), such as the default mode, executive control, and attention networks. The authors of Castellanos et al. (17) suggested that the long-range connections that link the dorsal anterior cingulate to the precuneus and posterior cingulate are a possible candidate locus of dysfunction in ADHD. Differently, the authors of Sripada et al. (18) proposed that the connectivity between the default mode and the ventral attention networks is a key locus of dysfunction in ADHD. In another study (19), altered intranetwork connectivity was observed in the default mode, dorsal attention, and visual networks. The study from Sun et al. (20) suggested that the development pattern of the interaction between the dorsal anterior cingulate and the default mode networks is abnormal in ADHD. The authors of Sutcubasi et al. (21) supported that ADHD can be characterized as an inter and intra default mode network dysconnectivity disorder. Aboitiz et al. (22) explored the connection between the dynamics of the catecholaminergic signaling in the brain and the activities of FBNs that regulate behavior. They identified abnormalities in the default mode, ventral attention, and salience networks, by exploring different lines of evidence, including pharmacology, brain imaging, and electrophysiology. Another study (23) that evaluated the functional connectivity of children with ADHD suggested that the dysregulation of FBNs in children with ADHD not only involves the dorsal attention and default mode networks but also the somatosensory, motor, visual, and auditory networks. Finally, regarding ADHD, it must be noted that several publications and research demonstrated language alterations in ADHD patients (24).

BD is characterized by manic, hypomanic, and major depressive episodes (1). According to the DSM-5 (1), in terms of symptomatology, family history, and genetics, bipolar and related disorders constitute a bridge between the SCZ spectrum and other psychotic and depressive disorders. Family history is one of the strongest and most consistent risk factors for BD. This psychiatric disorder leads to cognitive impairments and affects work performance, decreasing patients' quality of life. Vocational difficulties may also be present in BD individuals. Considering that some ADHD symptoms overlap with the symptoms of mania, it is possible to misdiagnose those psychiatric disorders (1).

Recently, Yoon et al. (25) did a review study on altered functional connectivity in BD. They suggested that the pathophysiology of BD is influenced by disrupted intranetwork and internetwork functional connectivity. The default mode, central executive, and salience networks, in particular, exhibit intranetwork hypoconnectivity. In addition, many of the studies that they reviewed demonstrated hyperconnectivity between the default mode and salience networks, while the relationship between the central executive and salience networks, and between the central executive and default mode networks indicated hypoconnectivity. Recently, Wang et al. (26) suggested that the salience and the basal ganglia networks play important roles in the dysfunctional emotional processing and regulation of BD patients. They also highlighted the role of the cerebellum, default mode, and sensory networks (sensorimotor, visual, and auditory networks) in the neuropathology of affective and cognitive deficits in BD. The default mode network can underline cognitive and affective processing problems and has a possible role in depressive relapses. The cerebellum plays a role in the regulation of emotion, affect, and cognitive processes, which are related to common symptoms in BD. Although abnormal motor and visual functions do not translate classical symptoms of BD, sensory networks also seem to play a role in the neuropathology of BD (26). Besides, several resting-state fMRI (rs-fMRI) studies in BD demonstrated abnormal ventral prefrontal cortex connectivity with the amygdala and other subcortical areas (thalamus, striatum) (27–29).

According to the DSM-5 (1), SCZ belongs to the SCZ spectrum and other psychotic disorders and results in significant social and occupational dysfunction. It is characterized by symptoms and signs in the following domains: delusions, hallucinations, disorganized thinking (speech), grossly disorganized or abnormal motor behavior (including catatonia), and negative symptoms (which include diminished emotional expression and avolition). In addition to the five characteristic symptoms, the assessment of cognition, depression, and mania symptom domains is crucial for differentiating between the various SCZ disorders of the spectrum. Genetic factors play a significant role in determining SCZ risk, and some risk alleles are shared by SCZ and other psychiatric disorders, such as BD. It must be noted that the distinction between SCZ and BD depends on the temporal relationship between the mood disturbance and the psychosis, and the severity of the depressive or manic symptoms, which sometimes lead to misdiagnosis (1).

Although the underlying causes and mechanisms of SCZ are uncertain, the hypothesis of functional brain disconnection is possible (30). Garrity et al. (31) compared the default mode network of SCZ patients with healthy subjects, and their results showed abnormalities in SCZ patients that correlated with both positive and negative symptoms. The salience networks seems also to be involved in SCZ (32). Additionally, Liang et al. (33) found decreased functional connectivity between the insula and the prefrontal lobe, the temporal lobe, and the corpus striatum, demonstrating that those regions may play an important role in the pathophysiology of SCZ. They also found increased connectivity between the cerebellum and other brain areas (33). The review study by Bernard et al. (34) provided further support for basal ganglia dysfunction in SCZ that may contribute to several symptoms, including cognitive deficits (34). Li et al. (35) found that SCZ patients and their first-degree relatives had similar disconnectivity patterns, even when the first-degree relatives did not present symptoms of the disorder. This was observed in the right executive control and ventral default mode networks. They also highlighted that hallucinations were found to be positively correlated with functional connectivity that links the left inferior frontal gyrus with the bilateral auditory cortex, right posterior temporal lobe, middle right anterior cingulate cortex, right ventral striatum, and left nucleus accumbens. Their study also showed hyperconnectivity between the bilateral thalamus and the language network, which may be associated with language, and consciousness abnormalities in patients with SCZ. Besides, functional connectivity between the higher visual and the right executive control networks was positively correlated with the severity of positive symptoms in SCZ. Additionally, they found abnormalities between the right executive control and the language networks, and between the precuneus system and the ventral default mode network (35). The authors of Skåtun et al. (28) also studied the functional connectivity of both SCZ and BD patients. Their study focused on thalamic regions. For SCZ, they found disrupted thalamic communication to the frontal lobe, possibly affecting higher-order cognitive processes. For both SCZ and BD, increased connectivity between thalamic and sensory regions was observed, which highlights the potential role of the thalamus on psychosis sensory disruptions (28).

In this study, we aim to investigate if normative models based on functional connectivity are able to discriminate against different psychiatric disorders (and address the heterogeneous aspect of such spectrum disorders). Therefore, we propose a normative model based on autoencoders to evaluate functional connectivity abnormalities in SCZ, BD, and ADHD. The underlying concept is that the autoencoder learns to copy the input data to its output. Therefore, the proposed autoencoder will learn to encode the healthy patterns from the input data to the latent representation, and then, only using the information from the latent representation, will try to reconstruct the input data as closely as possible to the original input data. As such, when presented with patient data it is expected that the reconstruction “fails,” and therefore abnormal functional connectivity patterns can be identified.

## 2. Materials and methods

### 2.1. Data description

The brain rs-fMRI images used in this project were obtained from three public neuroimaging databases. The Consortium for Reliability and Reproducibility (CoRR) public database (36) was chosen because it contains a large number of different datasets with data from healthy subjects, which is necessary for creating a generalized model of healthy subjects. The UCLA Consortium for Neuropsychiatric Phenomics LA5c Study (UCLA) public dataset (37) was selected to train and test the normative model since it contains healthy individuals and ADHD, BD, and SCZ patients. The Center for Biomedical Research Excellence (COBRE) public dataset (38–41), which is composed of healthy individuals and SCZ patients, was also chosen to evaluate the performance of the trained model on an independent dataset.

The original sample was reduced to a total of 700 subjects since several factors influence fMRI data, including the MRI scanner, scan parameters, age, and sex. The different scan equipment used by the various institutes results in a noticeable and unavoidable difference between the datasets. On the contrary, the scan parameters and the condition of the eyes during the scan contribute to data diversity but are more controllable variables. As a result, conditions were set up to ensure a large sample size while reducing the variability caused by those factors. We established an age range between 18 and 50 years old, a repetition time (TR) of 2 s, and we only included subjects who underwent the MRI scanner with their eyes open.

Table 1 presents additional information about the selected sample.

**Table 1:** Information about the data that were selected for the subsequent methods.

**Dataset**	**Number of subjects**	**Females (*%*)**	**Age range**	**Eyes condition**	**Manufacturer**	**Model**	**Headcoil**	**Field strength**	**Echo time (TE) [ms]**	**Repetition time (TR) [s]**	**Acquisition time (min:s)**	**Number of time points**	**Voxels (mm^3^)**
CoRR-BNU3	H-48	50	18–30	2	Siemens	TrioTim	12 channel	3T	30	2	8:06	240	3.5 × 3.5 × 3.5
CoRR-HNU1	H-30	50	20–30	1	GE	Discovery MR750	8 channel	3T	30	2	10:00	300	3.4 × 3.4 × 3.4
CoRR-IPCAS1	H-30	70	18–24	1	Siemens	TrioTim	8 channel	3T	30	2	6:54	205	4 × 4 × 4
CoRR-IPCAS3	H-34	65	18–25	1	Siemens	TrioTim	8 channel	3T	30	2	6:00	180	3.4 × 3.4 × 3
CoRR-IPCAS4	H-20	50	21–28	1	GE	Discovery MR750	8 channel	3T	30	2	6:04	180	3.5 × 3.5 × 3.5
CoRR-MRN1	H-39	59	18–49	1	Siemens	TrioTim	12 channel	3T	29	2	5:04	150	3.8 × 3.8 × 3.5
CoRR-NYU2	H-81	36	18–48	3	Siemens	Allegra	1 channel	3T	15	2	6:00	180	3 × 3 × 4
CoRR-Utah1	H-16	0	18–39	2	Siemens	TrioTim	12 channel	3T	28	2	8:06	240	3.4 × 3.4 × 3
CoRR-Utah2	H-1	0	39	2	Siemens	TrioTim	12 channel	3T	28	2	8:06	240	3.4 × 3.4 × 3
UCLA	H-130 ADHD-43 BD-49 SCZ-50	43	21–50	3	Siemens	TrioTim	NaN	3T	30	2	5:04	152	3 × 3 × 4
COBRE	H-74 SCZ-55	25	18–50	1	Siemens	TrioTim	NaN	3T	29	2	5:04	150	3.75 × 3.75 × 4.55

1, eyes open fixated on a target; 2, eyes open toward a blank screen; 3, eyes open; H, healthy; SCZ, schizophrenia; ADHD, attention-deficit hyperactivity disorder; BD, bipolar disorder; NaN, No information.

### 2.2. Data processing

First, brain extraction of the T1-weighted images of all subjects was performed with the OASIS template using the Advanced Normalization Tools (ANTs) (42). After finishing the preparation of the anatomical images, the pre-processing of rs-fMRI images was handled within the FMRIB Software Library (FSL) (43). We used the FSL's MELODIC tool (44) to perform several steps at once: (i) discard the first four volumes of functional data to ensure blood oxygen level-dependent (BOLD) fMRI signal stabilization; (ii) motion correction using the MCFLIRT tool, which attenuates the effect of head motion by spatially registering each volume separately to the middle volume, and estimates motion parameters; (iii) spatial smoothing using a Gaussian kernel with 5 mm full width at half maximum (FWHM) was also applied, which has the effect of blurring the images, and is achieved by calculating, at each voxel, a weighted average over the multiple voxels; (iv) high-pass filter with a cut-off of 0.01 Hz to remove frequencies lower than the low-frequency fluctuations that dominate the BOLD signal; (v) generate the necessary transformations to align the functional data to the standard space without performing registration; (vi) single-session spatial independent component analysis (ICA) with automatic dimensionality estimation was run, which allowed data to be unmixed into a set of independent components, each described by their time course and spatial map (45).

After running the FSL's MELODIC tool, it was possible to analyze the outputs of the MCFLIRT tool to evaluate the motion parameters of each subject. For defining general criteria for head motion rejection, the minimum value of each voxel size was found to be 3 × 3 × 3 mm^3^. Considering that, data in which the maximum framewise displacement exceeded 3 mm, motion in translation exceeded 3 mm, or angular deviation exceeded 3° were excluded from the sample (46, 47). Following these criteria, 32 subjects were removed from the analysis. In addition, it was not possible to finish data preprocessing for several subjects due to the lack of a rs-fMRI image or T1-weighted image.

In the end, 655 subjects continued to the subsequent analysis.

Spatial ICA was used for signal-denoising since this fully data-driven method estimates maximally independent components by selecting which ones explain most of the variance within the dataset. Considering that several generated components are not brain signals, those can be removed from the data. For that purpose, the FSL's tool FMRIB's ICA-based Xnoiseifier (FIX) was applied to automatically detect and remove non-brain signal components. To create a robust training set, ten healthy subjects from each dataset (and the subject from Utah2) were randomly selected, and hand-labeled classification of the ICA components was performed. The trained dataset was then used by FSL's tool FIX for classification and removal of the noise component of all subjects in the sample.

The cleaned 4D preprocessed functional image underwent registration to the individual T1-weighted structural image through the boundary-based registration (BBR) method, and then to the MNI152 standard space through a non-linear transformation.

For group-level comparisons, dual regression was applied. We used the fourteen FBNs from Shirer et al. (48) as a template [see Supplementary material for more information about the FBNs used in this study: anterior salience network (ASN), auditory network (AN), basal ganglia network (BGN), dorsal default mode network (DDMN), higher visual network (HVN), language network (LN), left executive control network (LECN), posterior salience network (PSN), precuneus network (PN), primary visual network (PVN), right executive control network (RECN), sensorimotor network (SN), ventral default mode network (VDMN), and visuospatial network (VN)]. Therefore, the registered functional data were used as input for deriving subject-specific maps. For the next steps, we only used the subject-specific time series, describing the temporal pattern of each FBN.

### 2.3. Functional brain connectivity

For investigating functional connectivity differences across individuals, a functional brain connectivity matrix was built for each subject. The Pearson's correlation coefficients between the time series of all FBNs pairs were calculated. In the end, a 14 × 14 functional connectivity matrix was created for each subject, giving information about inter FBNs connectivity. Considering that Pearson's correlation coefficient has no information about the direction of functional connectivity, the network matrix is symmetric. Therefore, to avoid redundancy, the diagonal matrix and one-half of the network matrix were discarded. Taking into account that $\frac{14\times 14-14}{2}=91$, the remaining half of the matrix was transformed into a vector containing 91 features (pairs of FBNs) for feeding the normative model.

### 2.4. Normative model

#### 2.4.1. Data split and standardization

The first step in the creation of the DL normative model was to split the data into different sets (see Table 2).

**Table 2:** Datasets for the normative model.

**Set group**	**Dataset**	**Number**	**Females (*%*)**	**Age range**	**Age mean**
H-Train	CORR and UCLA	366	47	18–49	26
H-Test-U	UCLA	39	51	21–50	34
SCZ-Test-U	UCLA	47	26	22–49	37
BD-Test-U	UCLA	45	44	21–50	35
ADHD-Test-U	UCLA	39	49	21–50	32
H-Test-C	UCLA	70	30	18–50	35
SCZ-Test-C	UCLA	49	18	19–50	31

Data were split into several sets of subjects. H-Train represents the 366 healthy subjects from CORR and UCLA datasets that were used to train the model. H-Test-U is the set of 39 healthy subjects from the UCLA dataset that were used to test the model. SCZ-Test-U, BD-Test-U, and ADHD-Test-U are the sets of psychiatric patients consisting of 47 SCZ patients, 45 BD patients, and 39 ADHD patients from the UCLA dataset, respectively. H-Test-C and SCZ-Test-C are the independent test sets consisting of 70 healthy subjects, and 49 SCZ patients from the COBRE dataset, respectively.

Data standardization was performed before training the model.


(1) $$\begin{array}{l} z=\frac{x-\mu }{\sigma } \end{array}$$

where x represents the original value of the feature for a specific subject, μ represents the mean value of the feature for all subjects of the H-Train set, and σ the standard deviation value of the feature for all subjects of the H-Train set.

To calculate the mean and the standard deviation values for standardization, only the subjects belonging to the training set were used. After that, standardization was applied to all sets of subjects.

#### 2.4.2. Model training

Autoencoder was the selected architecture for the generation of the normative model, and it was implemented using the *Keras* Python library. For optimization, only the training set fed the model, to avoid bias in the analysis. Ten fold cross-validation was applied to validate the optimized model while studying its generalization. The proportion of datasets was kept similar for the different folds. The model was trained and tested ten times, using a different fold as the validation set in each time. For optimizing the model, a global metric per subject was defined by calculating the mean squared error between the reconstructed data and the input data of the autoencoder for each subject of the different sets (*MSE*_*s*_):


(2) $$\begin{array}{l} MSE_{s}=\frac{1}{n}\overset{n}{\underset{i=1}{\sum }}{\left(ŷi-yi\right)}^{2} \end{array}$$

where *n* is the number of features (91), ŷ*i* is the reconstructed feature *i*, and *yi* is the inputted feature *i*.

The mean of the median *MSE*_*s*_ values of the training set for the 10 folds of cross-validation, and the difference between the mean of the median *MSE*_*s*_ values of the training set and the mean of the median *MSE*_*s*_ values of the validation set for the 10 folds of cross-validation were calculated for optimizing the model. Those metrics were used to evaluate the performance and the generalization of the model on reconstructing the data, respectively. Considering that a conjugation between both metrics is crucial to decide which one is the best model architecture, and by knowing that the range of their values was different, both metrics were normalized.


(3) $$\begin{array}{l} x_{norm}=\frac{x_{original}-min}{max-min} \end{array}$$

where *x*_*norm*_ is the normalized value of the evaluation metric, *x*_*original*_ is the original value of the evaluation metric, and *min* and *max* are the minimum, and maximum values of the evaluation metric, respectively.

The model that better balanced both normalized metrics was selected for training. It consisted of three hidden layers (91–46–13–46–91), with the activation function of the hidden layers being the leaky rectified linear unit. A L2 regularization parameter of 1^−5^ and a dropout value of 0.5 were applied. Additionally, the mean squared error was chosen as the loss function, the weights were initialized following He initialization, and the model was trained for 1,000 epochs, using Adam optimization (β1 = 0.9, β2 = 0.999, ϵ = 10^−7^, and a learning rate of 0.0005).

#### 2.4.3. Model evaluation

After training the autoencoder with 366 healthy subjects, it was tested with both healthy subjects and patients.

The global metric per subject defined in Equation 2 was used to generate a boxplot for each test group, and the median values were compared between the different test sets. Besides, a Mann–Whitney statistical test was applied between the *MSE*_*s*_ values of different groups of subjects to evaluate if the differences between groups were statistically significant. Additionally, the absolute difference between the output vector and the input vector was calculated. The generated difference vectors for each subject were reshaped into a 14 × 14 lower triangular matrix, and binarized using the threshold that is defined in the results section. Note that the retained values represent pairs of FBNs that were more abnormal. Graph theory was used to obtain the network degree for each subject. The number of degrees represents the number of connections to a node. For each test group, the median value of the number of degrees was calculated for each FBN. In the end, for each test group, a 14-row vector was generated, representing the median value of the number of degrees for each FBN.

To study which pairs of FBNs were abnormal in each test set, the mean squared error was calculated for each feature, giving a global metric per feature [mean squared error per feature (*MSE*_*f*_)].


(4) $$\begin{array}{l} MSE_{f}=\frac{1}{N}\overset{N}{\underset{i=1}{\sum }}{\left(\hat{f}i-fi\right)}^{2} \end{array}$$

where *N* is the number of subjects in each test set, $\hat{f}i$ is the reconstructed feature of subject *i*, and *fi* is the inputted feature of subject *i*.

For each test set, the *MSE*_*f*_ values of each pair of FBNs were combined into a matrix.

To determine which pairs of FBNs were specific of each group of patients, the *MSE*_*f*_ matrix of healthy subjects (H-Test-U set) was subtracted from the *MSE*_*f*_ matrices of each group of patients. To more robustly investigate the pairs of FBNs that characterize each group of patients, an additional analysis with a larger H-Test-U set was conducted (see Supplementary material).

Finally, the pairs of FBNs characteristic of each group of patients were analyzed for each subject, to assess whether the group-level results were still present at the individual-level.

#### 2.4.4. Schizophrenia case study

In the end, the normative model was tested on an independent dataset, COBRE. This way it was possible to compare the results of SCZ for the UCLA and COBRE datasets, in order to evaluate the generalization of the model.

## 3. Results

### 3.1. Analysis of the reconstruction error per subject

The *MSE*_*s*_ were calculated for each subject, and boxplots were generated for each group of subjects.

**Figure 1:**
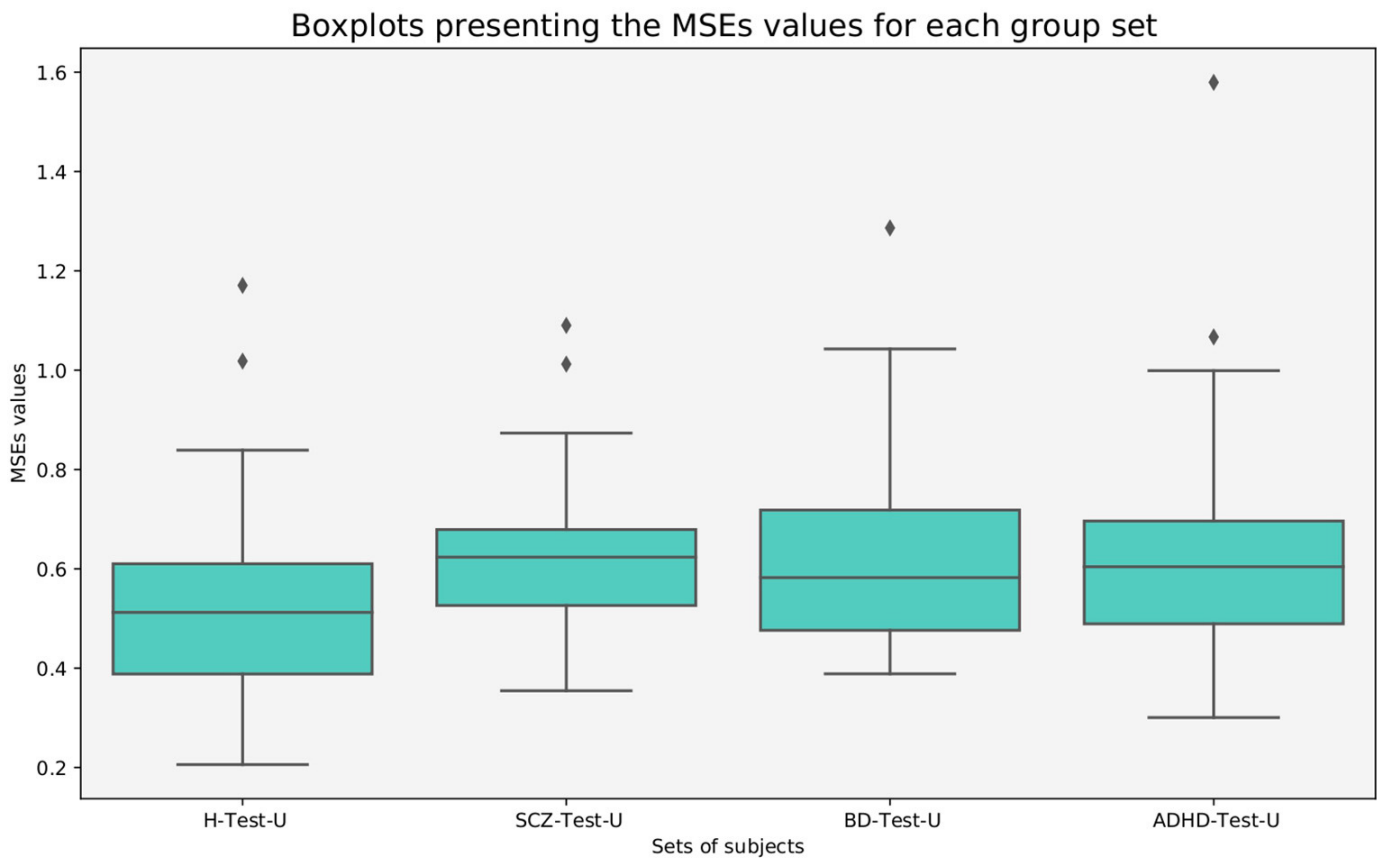
Boxplots presenting the *MSE*_*s*_ values of all subjects, for each group.

From Figure 1 it is evident that the healthy test set presents a lower median *MSE*_*s*_ value than all patient test sets, suggesting that the model was able to learn healthy specific characteristics.

A Mann–Whitney test was selected to evaluate if the differences between groups were statistically significant, since the Shapiro–Wilk test rejected the null hypothesis that the data was normally distributed for all groups of subjects presented in Figure 1 (see Table 3).

**Table 3:** Mann–Whitney test between the *MSE*_*s*_ values data of the groups of subjects.

**Group I**	**Group II**	***p*-value**
H-Test-U	SCZ-Test-U	0.002
H-Test-U	BD-Test-U	0.011
H-Test-U	ADHD-Test-U	0.007
SCZ-Test-U	BD-Test-U	0.339
SCZ-Test-U	ADHD-Test-U	0.452
BD-Test-U	ADHD-Test-U	0.373

Considering a *p* = 0.05, it is noticeable that the differences between the groups of patients and the healthy subjects are statistically significant. On the contrary, the null hypothesis is not rejected for the statistical test between the groups of patients. These findings show that the normative model was able to learn healthy characteristics and distinguish patients from healthy individuals.

### 3.2. Analysis of the reconstruction error per pair of functional brain networks

#### 3.2.1. Graph theory metrics calculation

**Figure 2:**
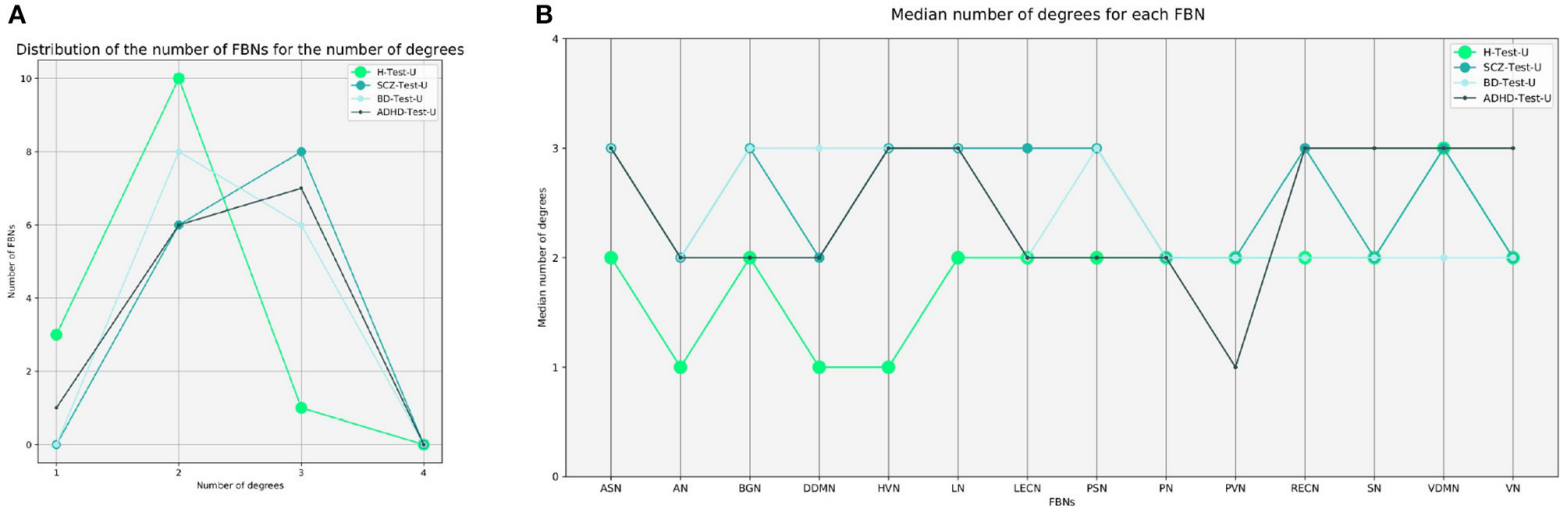
Graph theory analysis. **(A)** Distribution of the number of FBNs for the number of degrees, for the H-Test-U, SCZ-Test-U, BD-Test-U, and ADHD-Test-U groups. **(B)** Representation of the median number of degrees for each FBN, for the H-Test-U, SCZ-Test-U, BD-Test-U, and ADHD-Test-U groups. FBNs, functional brain networks; ASN, anterior salience network; AN, auditory network; BGN, basal ganglia network; DDMN, dorsal default mode network; HVN, higher visual network; LN, language network; LECN, left executive control network; PSN, posterior salience network; PN, precuneus network; PVN, primary visual network; RECN, right executive control network; SN, sensorimotor network; VDMN, ventral default mode network; VN, visuospatial network.

Graph theory was also applied to perform group analysis, by transforming the difference vectors into binarized matrices. For defining the threshold, we first merged the difference vectors of all subjects used to test the autoencoder. Then, we considered the 20% higher values of the merged vector, which corresponds to a threshold value equal to 1. The elements of the matrix that were retained represent the pairs of FBNs that were worse reconstructed regarding network degree of the difference in reconstruction, which are those that are probably functionally abnormal. Figure 2A shows the number of FBNs distributed by the median number of degrees. It is observed that the sets of the patients are right-shifted when compared with the healthy subjects. In comparison to healthy people, SCZ, BD, and ADHD patients have a greater number of connected FBNs. This means that there are more pairs of FBNs worse reconstructed for the test sets of patients.

The median number of degrees were also regionally analyzed. Figure 2B displays the median number of degrees for each FBN. The FBNs with the most number of degrees are the ones that were poorly reconstructed. Considering that some FBNs have more degrees than others, not all FBNs are reconstructed in the same way. The ASN, HVN, LN, RECN, SN, VDMN, and VN are the more abnormal FBNs in the ADHD group. The ASN, BGN, DDMN, HVN, LN, and PSN are the more atypical FBNs in the BD group. The ASN, BGN, HVN, LN, LECN, PSN, RECN, and VDMN are the more divergent FBNs in the SCZ group. The results demonstrate that different psychiatric disorders have different characteristics and that the normative model can determine the FBNs that are more abnormal for each group. Nevertheless, even the group of healthy subjects present some FBNs that are worse reconstructed than others. This may be justified by the fact that some FBNs are more variable among healthy subjects than others, and that their functional connectivity was more difficult to learn by the normative model. Even though, it must be noted that threshold decision influences the results.

#### 3.2.2. *MSE* calculation for each pair of functional brain networks

**Figure 3:**
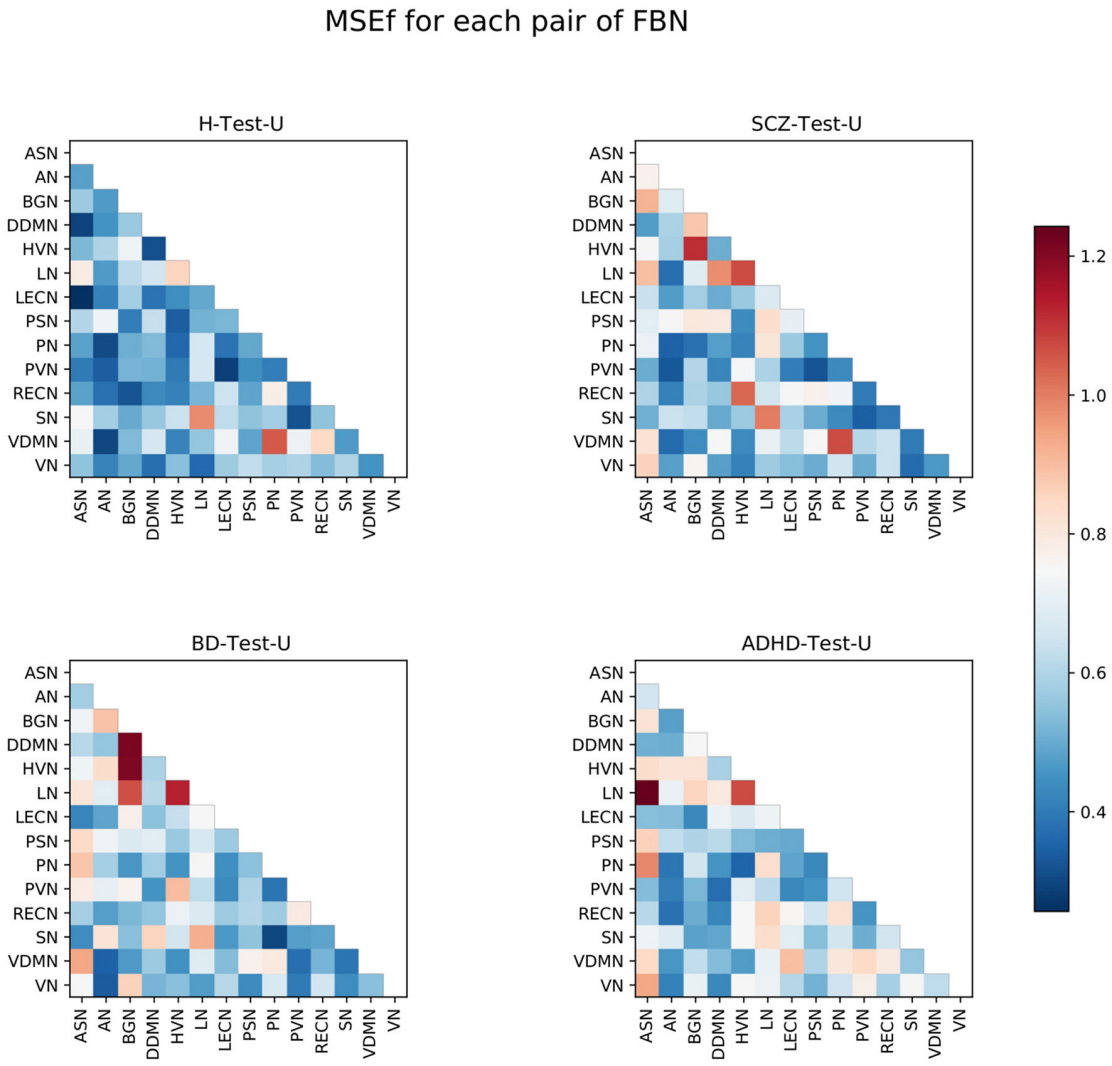
Matrices representing the *MSE*_*f*_ for each pair of FBNs, for the four test sets. The color bar ranges between the lowest and the highest values from the *MSE*_*f*_ for each pair of FBNs of all test sets.

Afterward, the *MSE*_*f*_ was calculated for each pair of FBNs. The resulting matrices for each set of subjects are presented in Figure 3.

The results are consistent with the hypothesis that the normative model might not reconstruct every pair of FBNs similarly. Some pairs of FBNs appear to be more difficult to reconstruct than others.

The pairs LN-HVN, SN-LN, and VDMN-PN are worse reconstructed for the H-Test-U, and are also highlighted for the other three test sets. This suggests that the functional connectivity of those pairs of FBNs varies even among healthy subjects. In addition, some FBNs present more abnormal connectivity than others, for each test set. This is consistent with the results from graph theory analysis since most of the pairs of FBNs that are highlighted in Figure 3 involve the FBNs that were considered to be atypical following graph theory analysis for each test set (Figure 2B). For ADHD, several pairs of FBNs that were highlighted involved the ASN. For BD, the BGN and the LN are the FBNs that are more evidently different. For the SCZ, the BGN, HVN, and LN are the FBNs that are more noticeable. This corroborates some of the findings of the graph theory analysis, since the two different methods led to similar results.

**Figure 4:**
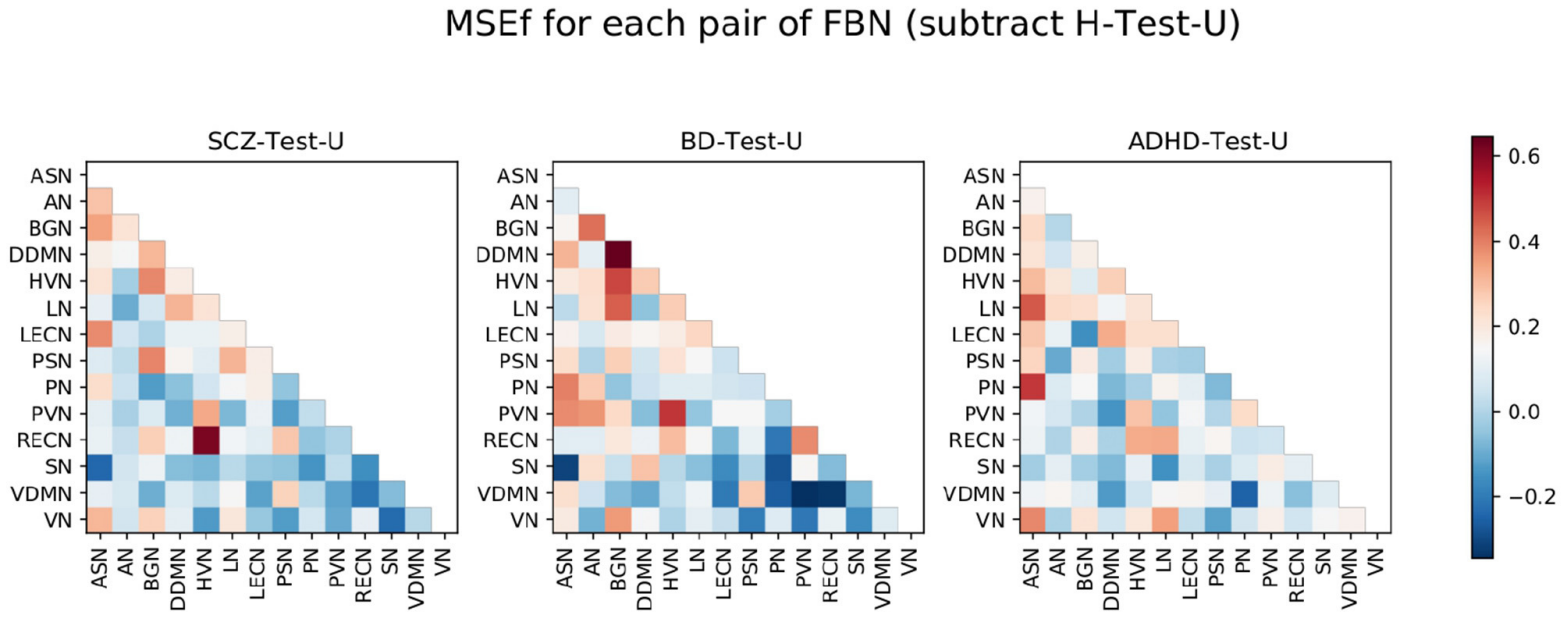
SCZ-Test-U, BD-Test-U, and ADHD-Test-U matrices represented in Figure 3, subtracted by the H-Test-U matrix. The color bar ranges between the lowest and the highest value from the *MSE*_*f*_ for each pair of FBNs of all test sets after subtraction of the H-Test-U *MSE*_*f*_ matrix.

Considering that the H-Test-U matrix also contains elements with a high *MSE*_*f*_, the *MSE*_*f*_ matrix of the healthy subjects was subtracted to each of the other *MSE*_*f*_ matrices from the groups of patients (Figure 4). Thus, the pairs of FBNs that are highlighted represent abnormalities that may be specific to that group of patients, since a group of healthy subjects from the same dataset is used to exclude pairs of FBNs that are variable even among healthy individuals.

**Figure 5:**
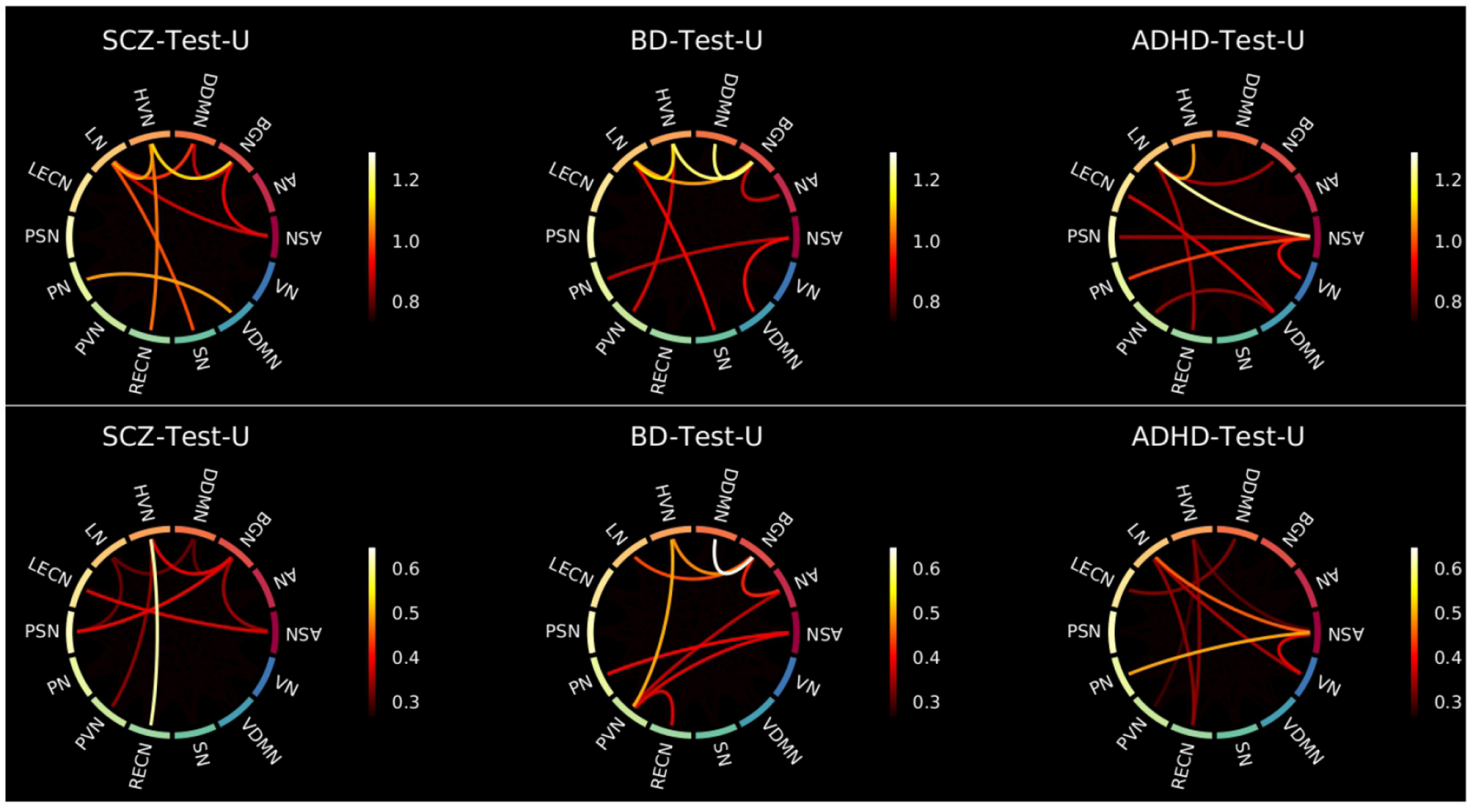
Connectograms showing the 10% of pairs of FBNs that were worse reconstructed for each group without (top) and with (bottom) subtraction of the H-Test-U *MSE*_*f*_ matrix. The color bar ranges between the lowest and highest values of those 10% of pairs of FBNs extracted from Figures 3, 4, respectively.

To analyze those results in detail, Figure 5 was displayed. It shows the 10% of worst reconstructed pairs of FBNs for each test, before and after subtracting the *MSE*_*f*_ matrix of healthy subjects.

#### 3.2.3. Functional brain networks that characterize each group of patients

To investigate more robustly which pairs of FBNs characterize each group of patients, an additional analysis with a larger H-Test-U set was conducted. This was done since the H-Test-U set was used to test the ability of the algorithm to learn specific healthy characteristics and, consequently, the ability to characterize the different patient groups (see Supplementary material).

For this additional analysis, it was decided to double the size of the H-Test-U. Therefore, two conditions may be taken into account in analyzing the results: (a) a model with a H-Test-U set with 39 healthy individuals and a corresponding H-Train set with 366 healthy individuals; (b) a model with a H-Test-U set with 78 healthy individuals and a corresponding H-Train set with 327 healthy individuals.

Besides, for a better understanding of the results, it should be noted that: (1) the 10% worse reconstructed pairs of FBNs that were highlighted without and with subtraction of the H-Test-U should be considered characteristic of that group of psychiatric patients since they were easy to reproduce for the healthy subjects. (2) The 10% worse reconstructed pairs of FBNs that were highlighted before subtracting the *MSE*_*f*_ matrix of healthy subjects but were not present after subtraction are not useful to distinguish the group of patients from the group of healthy subjects. They probably do not present a stable connectivity pattern even among healthy individuals and may not characterize that patient group. On the contrary, they may be specific to that group of patients, and have been poorly reconstructed due to model limitations, which are presented later in the discussion section. (3) The 10% worse reconstructed pairs of FBNs that were not highlighted before the subtraction, and were present after, are those that were easy to learn by the normative model, indicating that they are stable among healthy people, but are probably abnormal in the group of patients. Considering that, the pairs of FBNs that are considered to characterize the patient groups need to follow rules (1) or (3) for conditions (a) and (b).

For the SCZ-Test-U, the correlations between the ASN-BGN, BGN-HVN, and HVN-RECN are pairs of FBNs that characterize the group, since they are present before and after subtraction of the *MSE*_*f*_ matrix of the healthy subjects for both conditions. The ASN-LECN, BGN-PSN, and HVN-PVN pairs of FBNs also characterize the SCZ group following rule (3). This suggests that the connectivity pattern between these FBNs is important to characterize the healthy pattern, and that the normative model tried to minimize the reconstruction error of those features. Thus, for the SCZ-Test-U, the reconstruction error of those pairs of FBNs was not very high, because the normative model strives to reconstruct those features well. On the contrary, the other pairs of FBNs that were not mentioned above might not accurately describe the SCZ-Test-U, because the normative model failed to successfully learn how to reconstruct those features for healthy people or because different conclusions were drawn for conditions (a) and (b).

For the BD-Test-U, the BGN-DDMN, BGN-HVN, BGN-LN, HVN-PVN pairs of FBNs were kept before and after subtraction of the *MSE*_*f*_ matrix of the healthy subjects for conditions (a) and (b), and consequently, they should characterize the BD group. The AN-BGN, AN-PVN, and PVN-RECN are also characteristic of BD patients since they were highlighted after subtracting the matrix of the healthy test subjects for conditions (a) and (b). On the contrary, the other pairs of FBNs that were not mentioned above should not be specific of the BD-Test-U set.

The ASN-LN, ASN-PN, and LN-RECN pairs of FBNs are representative in the ADHD-Test-U. In addition, the HVN-PVN, ASN-VN and LN-VN also seem to characterize the ADHD group, because their reconstruction error is distinguishable from the reconstruction error of the healthy subjects. Differently, for the other pairs of FBNs that were not mentioned above, it is not possible to conclude that their functional connectivity pattern is useful to characterize the ADHD group.

#### 3.2.4. Individual-level analysis of the pairs of FBNs that characterize each group of patients

**Figure 6:**
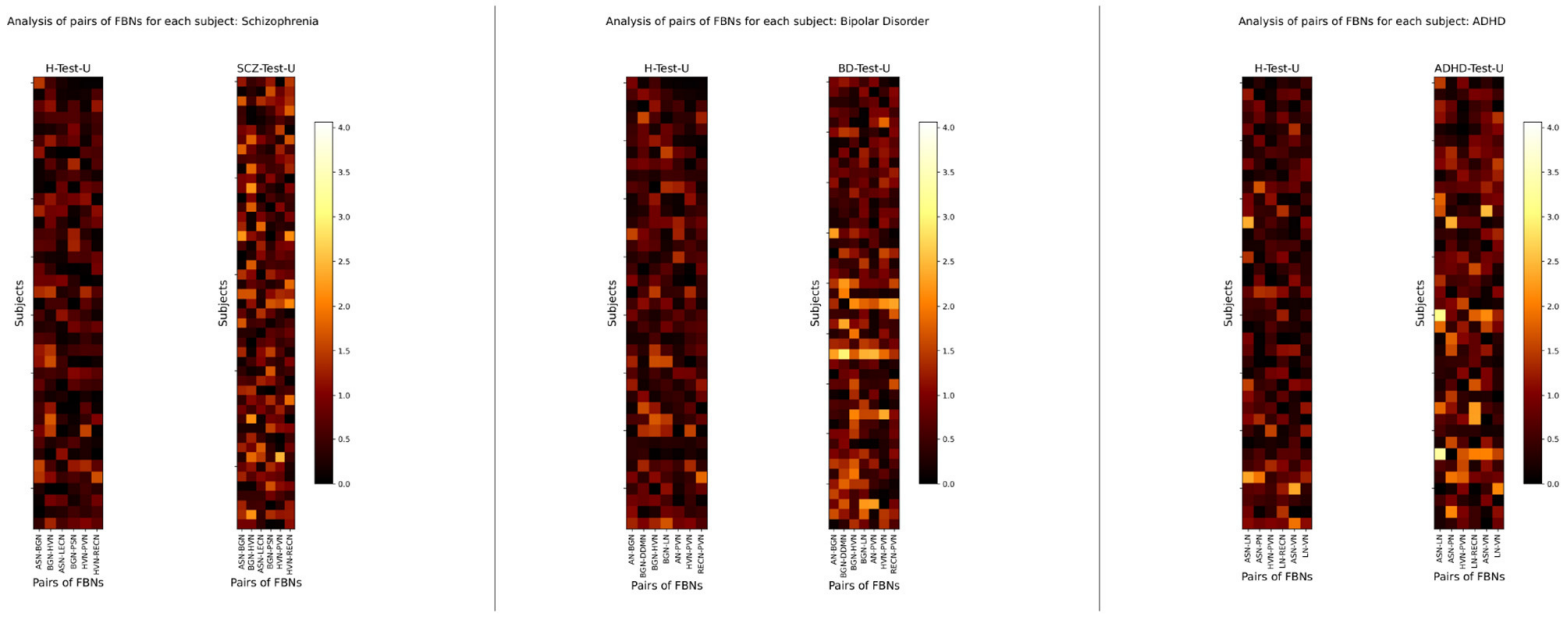
Individual-level analysis of the absolute values of the differences between the reconstructed and inputted data of the pairs of FBNs that characterize SCZ-Test-U, BD-Test-U, and ADHD-Test-U groups. The color bar ranges between the lowest and the highest values of the absolute difference between the reconstructed and inputted data for the selected features of all subjects used for testing the normative model.

After performing the group-level analysis, a simple representation of the individual patterns was conducted to determine whether the group findings persisted across all subjects. To this end, an individual-level analysis of the pairs of FBNs that were previously considered characteristic of each group of patients was done. Figure 6 shows the absolute values of the differences between the reconstructed and inputted data for those pairs of FBNs, for each subject, for the H-Test-U, SCZ-Test-U, BD-Test-U, and ADHD-Test-U sets.

The findings show that SCZ, BD, and ADHD patients generally have larger reconstruction errors than healthy subjects, although this trend varies among individuals. Besides, high heterogeneity is found within the same set of patients, which suggests that the group-level findings may not resist an individual-level analysis.

### 3.3. Schizophrenia case study

**Figure 7:**
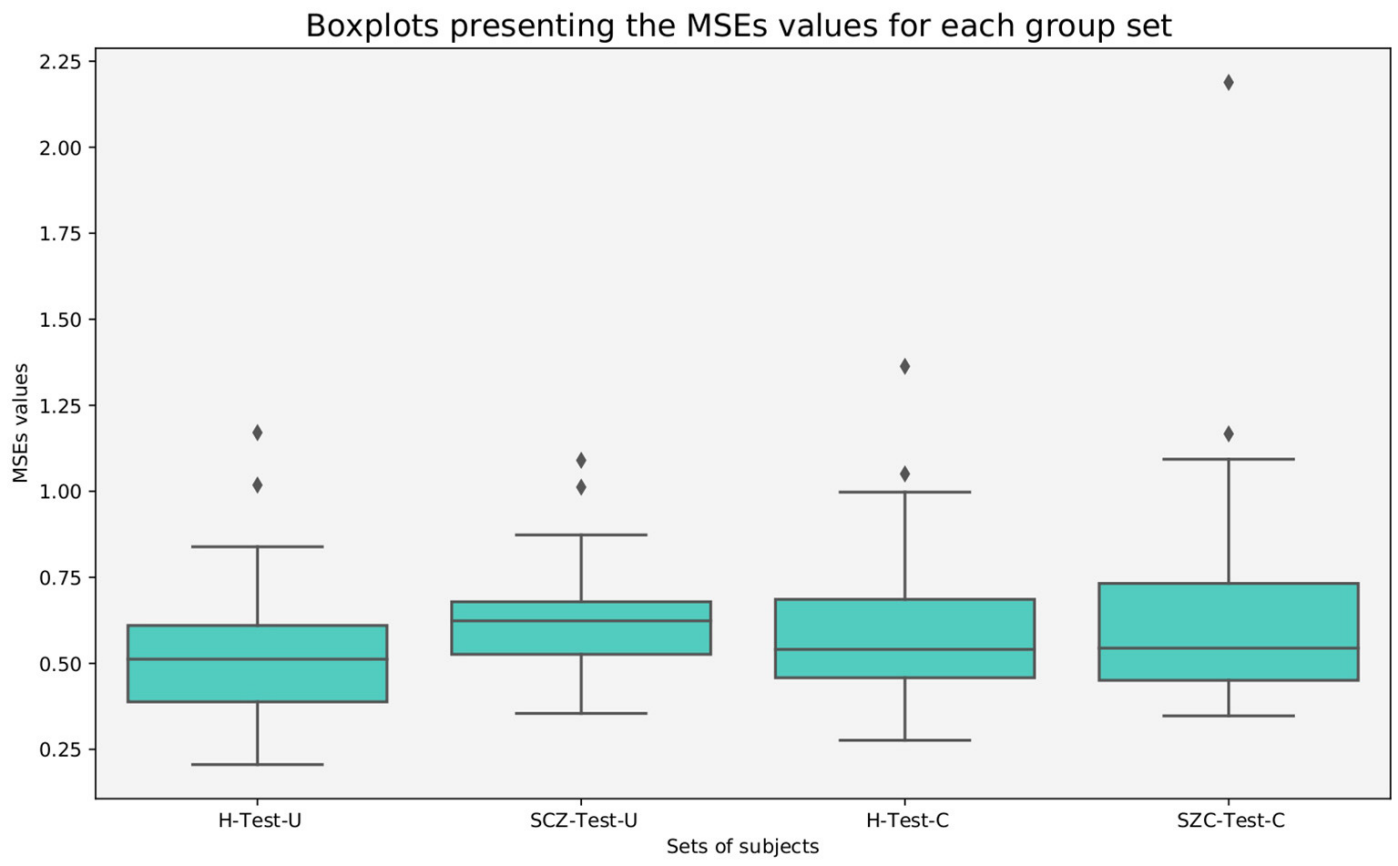
Boxplots presenting the *MSE*_*s*_ values of all subjects, for healthy and SCZ subjects from the UCLA and COBRE datasets.

The trained autoencoder was tested with an independent test set. The healthy subjects and the SCZ patients from the COBRE dataset were used to evaluate the generalization of the model. Therefore, to compare the results, those are presented in combination with the results from the H-Test-U, and SCZ-Test-U. Figure 7 displays the boxplots of the *MSE*_*s*_ values. The differences between the boxplots of H-Test-C and SCZ-Test-C are not as notorious as those from the UCLA dataset. Nevertheless, the SCZ-Test-C boxplot is shifted up when compared to the H-Test-C boxplot, which may indicate that the normative model has more difficulty in reconstructing data from the SCZ patients. For the COBRE dataset, the Shapiro-Wilk test also rejected the null hypothesis that the data were normally distributed. Thus, the Mann-Whitney test was applied (Table 4)

**Table 4:** Mann-Whitney test between the *MSE*_*s*_ values data of the groups of subjects, for the COBRE dataset.

**Group I**	**Group II**	***p*-value**
H-Test-U	H-Test-C	0.100
H-Test-C	SCZ-Test-C	0.324

**Figure 8:**
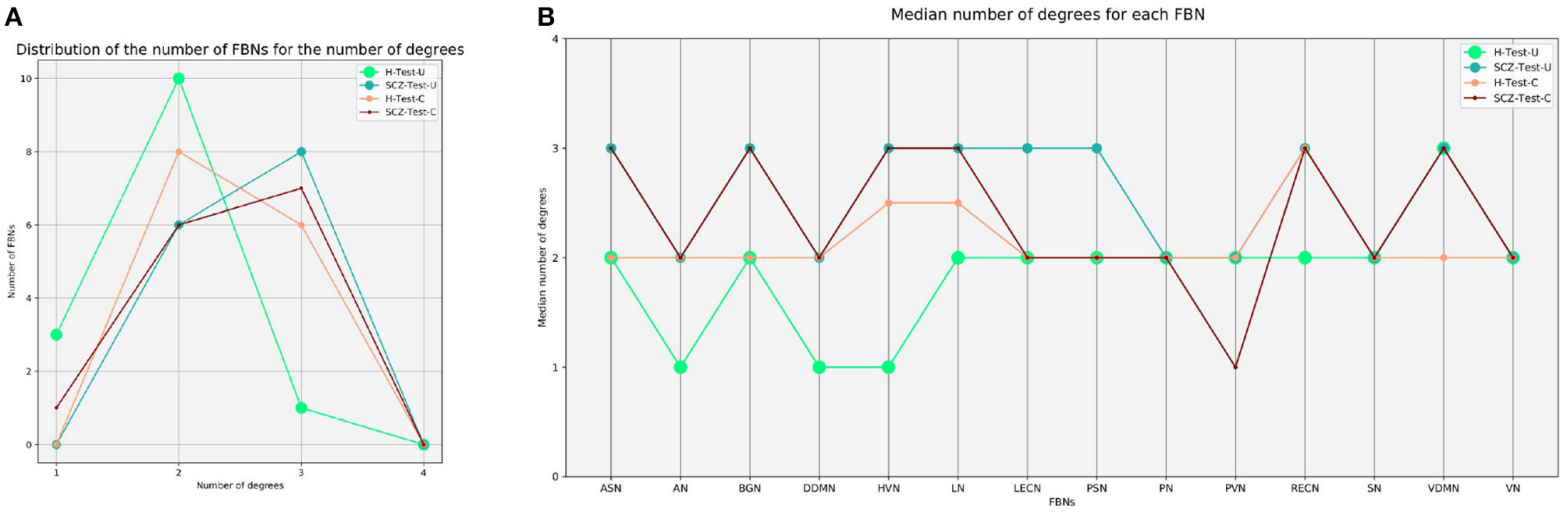
Graph theory analysis for the COBRE dataset. **(A)** Distribution of the number of FBNs for the number of degrees, for the H-Test-U, SCZ-Test-U, H-Test-C, and SCZ-Test-C groups. **(B)** Representation of the median number of degrees for each FBN, for the H-Test-U, SCZ-Test-U, H-Test-C, SCZ-Test-C groups.

Considering a *p* = 0.05, the null hypothesis is not rejected for the comparison between the groups of healthy subjects. When comparing the both groups of the COBRE dataset, the differences between the SCZ patients and the healthy individuals are not statistically significant. This suggests that the model's ability to distinguish healthy individuals from patients in an independent dataset is less accurate. Figure 8A shows that the distribution of the number of FBNs by the median number of degrees is similar for both sets. Nonetheless, it seems that the H-Test-C contains less abnormal FBNs than the SCZ-Test-C.

Figure 8B shows some distinct differences, by examining each FBN separately. Both groups of SCZ patients have similar patterns, which suggests that the model can characterize SCZ. On the contrary, there are fewer similarities between the patterns of both groups of healthy subjects.

**Figure 9:**
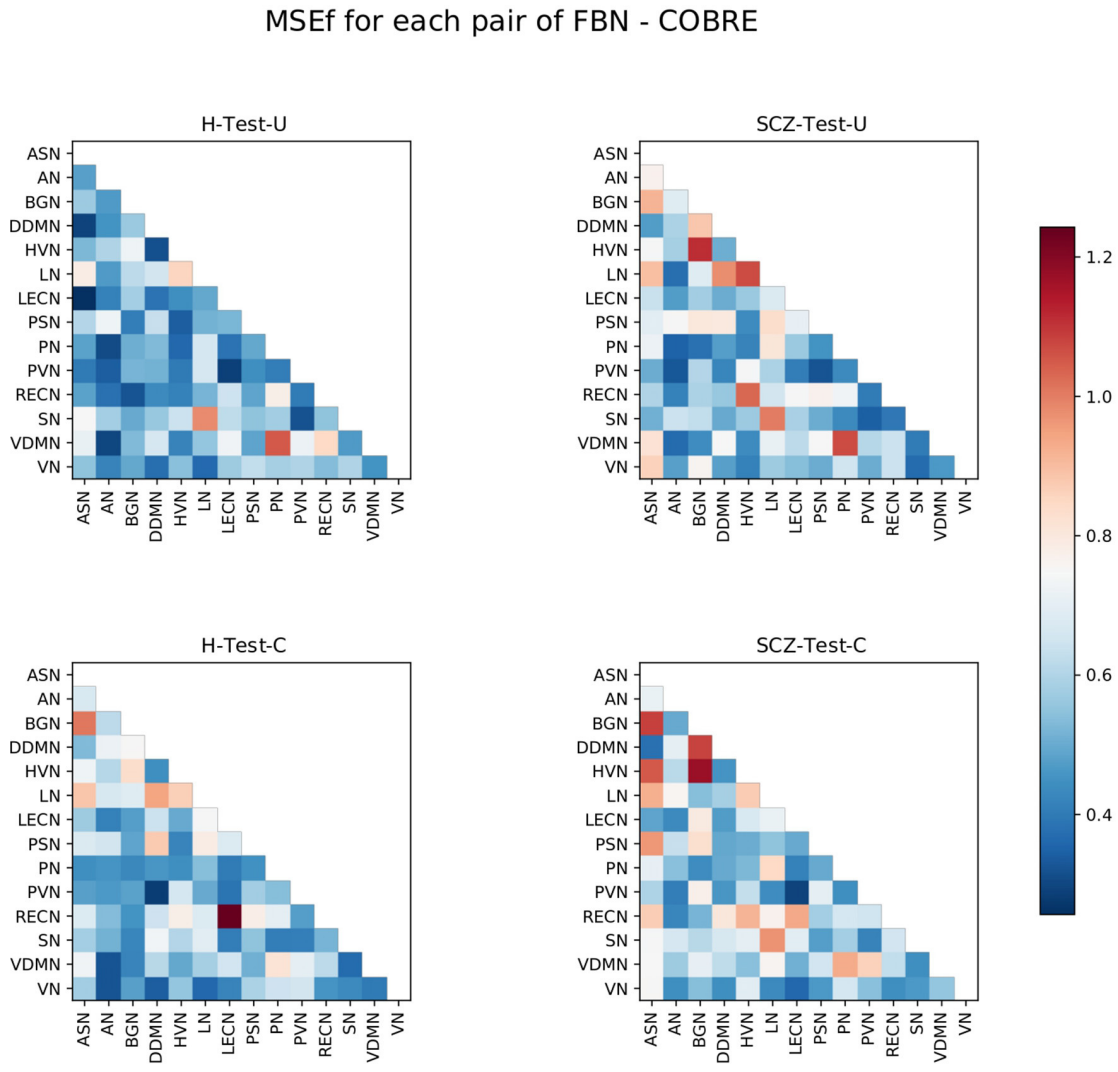
Matrices representing the *MSE*_*f*_ for each pair of FBNs, for the healthy **(Left)** and SCZ **(Right)** test sets of the UCLA **(Top)** and COBRE **(Bottom)** datasets. The color bar ranges between the same values of Figure 3.

Analyzing the connectivity between the pairs of FBNs, it is possible to deeper explore those findings. Figure 9 shows that in general the pairs of FBNs that were worse reconstructed for the H-Test-C have a lower reconstruction error than those from the SCZ-Test-C and SCZ-Test-U. In addition, both SCZ groups have a similar pattern, with a high reconstruction error for many of the same pairs of FBNs. This indicates that the normative model was successful in identifying characteristics of SCZ. Differently, the two groups of healthy subjects do not present such similar patterns. The pairs of FBNs shared between those healthy groups that were poorly reconstructed may represent patterns of connectivity that are not stable in healthy individuals (e.g., the ASN-LN, and the PN-VDMN). Other pairs of FBNs that appear in one of the healthy groups but not the other could be associated with the specific dataset. For example, the pair LECN-RECN seems to be unique to the COBRE dataset, as it is highlighted in both COBRE groups, but not in any of the other test groups.

**Figure 10:**
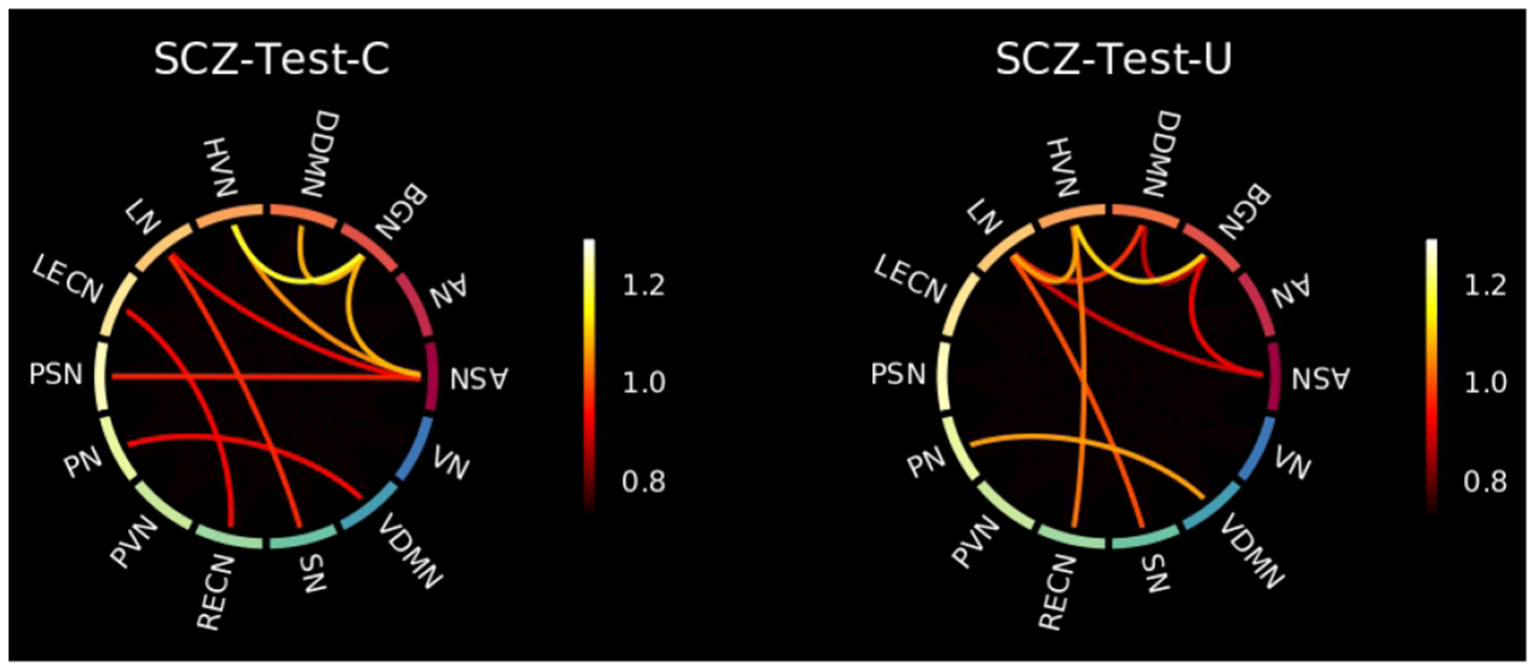
Connectograms showing the 10% of pairs of FBNs that were worse reconstructed for the SCZ patients of the COBRE **(Left)** and UCLA **(Right)** datasets. The color bar ranges between the lowest and highest values of those 10% of pairs of FBNs extracted from Figure 9.

Figure 10 shows the SCZ patients' connectograms from the UCLA and COBRE datasets. From the nine thresholded pairs of FBNs (10% of pairs of FBN worse reconstructed), six are shared between the two groups. These incude the ASN-BGN, BGN-HVN, BGN-DDMN, ASN-LN, LN-SN, and PN-VDMN. Besides, the ASN-BGN and BGN-HVN were also considered characteristics of the SCZ-Test-U set. Nonetheless, it must be observed that the *MSE*_*f*_ of some of those shared pairs of FBNs are also high in the groups of healthy subjects. On the contrary, the DDMN-LN, HVN-RECN, HVN-LN, ASN-HVN, LECN-RECN, ASN-PSN are not thresholded for the two groups of SCZ patients, although some of these pairs of FBNs still have a high *MSE*_*f*_ value. Overall, this suggests again that the normative model was able to identify SCZ patterns.

## 4. Discussion

In this study, we evaluated the performance of a normative model on rs-fMRI data of SCZ, BD and ADHD patients. First, a group-level analysis was pursued for evaluating if the developed normative model would be able to identify disorder's patterns in agreement with the literature. A simple individual-level analysis was also done to evaluate the heterogeneity within each group of patients.

Our results demonstrate that patients deviate more than healthy individuals from the pattern learned by the normative model (Figure 1). This was further supported by the graph theory approach, which demonstrates that patient groups show more FBNs with a higher number of degrees (Figure 2). Besides, the abnormal pairs of FBNs that seem to characterize each group are in agreement with several literature findings (Figures 3–5).

According to the literature, the default mode and salience networks appear to play a role in SCZ, which is consistent with the findings of this study, especially given the match between the FBNs of Shirer et al. (48) and the FBNs of Thomas Yeo et al. (49) reported in the Supplementary material. Considering that the anterior and posterior insula are located in the ASN and PSN, respectively, that corpus striatum belongs to basal ganglia, and that the LECN combines regions of the temporal and prefrontal lobes, the pairs of FBNs corresponding to the ASN-BGN, BGN-PSN, and ASN-LECN that were highlighted in the SCZ-Test-U follow the findings of Liang et al. (33), previously mentioned in the Introduction. The role of the basal ganglia in SCZ was previously reported (34), and is also found in the results of this study. The abnormal connectivity between the HVN and the RECN was also reported in previous studies (35), and that pair of FBNs was the most abnormal in this group of SCZ patients when compared with the group of healthy subjects.

The role of the BGN in BD is evident from the results and is in agreement with some studies in the literature. It has been previously suggested that basal ganglia may play an important role in the dysfunctional emotional processing and regulation of BD patients (26). Nevertheless, whereas in this study the BGN is highlighted as the FBN that is more functionally abnormal in BD, in the literature it has been demonstrated that some other FBNs appear to be more relevant in BD, such as the default mode, central executive, and salience networks (25). Our results demonstrate that the ASN seems to be abnormal in BD, but we did not find any significant abnormalities related to executive control networks in our study. There are parts of the default mode network (DDMN, PN, and LN) that seem to be abnormal in BD, but they do not seem to play a role as relevant as the BGN. Unfortunately, the cerebellum network was not included in this study, and it also seems to play a role in BD (26). The thalamus was also previously considered to play a role in BD, which may justify the atypical functional connectivity of the BGN since it includes thalamic regions. In particular, the abnormal connectivity between the DDMN and BGN is in line with the studies that mentioned the atypical connectivity between the ventral prefrontal cortex and basal ganglia regions (27–29). The role of the visual regions, which include the PVN and the HVN, also characterize BD in the present study. Although visual regions have been previously reported to be related to the neuropathology of BD, not much focus has been given to FBNs related to visual processes (26).

In ADHD, the pair ASN-PN presents the highest *MSE*_*f*_ among the ADHD group, which corroborates with a previous study that considered the link between the dorsal anterior cingulate and the precuneus to be a possible candidate locus for the dysfunction of ADHD (17). The abnormal interplay of the default mode, executive control and attention networks (16) in ADHD was also observed in this study, considering that the LECN and the RECN make up the executive control networks, the ASN and the VN are the attention networks, and the DDMN and the LN belong to the default mode network. All those FBNs were found to be involved in the functional connectivity pattern of ADHD. Additionally, the visual networks (HVN and PVN) also seem to be involved in the neuropathology of ADHD. This is in line with a recent study (23), which highlighted the role of somatosensory FBNs in ADHD. The language alterations reported in ADHD patients are also in line with the results since the LN seems to be functionally abnormal in ADHD (24).

Several results are in agreement with the literature, which demonstrates the ability of the normative model to identify functional connectivity patterns of several psychiatric disorders.

The good performance of the normative model is also supported by the results obtained for the independent dataset, COBRE. A similar pattern for both groups of SCZ patients is observed in Figure 9, which demonstrates that the normative model was able to identify characteristics of SCZ. Overall, the results obtained for the COBRE dataset suggest that the normative model was generalizable.

Regarding the individual-level analysis, the results presented in Figure 6 show that, although generally, the reconstruction error of the patients is higher than the reconstruction error of the healthy subjects, this is not true for all subjects. Besides, the individual pattern of reconstruction errors of each patient is not constant among all individuals within the same group. Those results are expected and are in line with the premise of this study, which is that psychiatric disorders comprise a spectrum with undefined boundaries leading often times to misdiagnosis.

As an example, the characteristic symptoms of SCZ involve a range of cognitive, behavioral, and emotional dysfunctions. However, no single symptom is specific to the disorder. SCZ diagnosis involves the recognition of a constellation of signs and symptoms associated with occupational or social impairments. Therefore, SCZ can be considered a heterogeneous psychiatric disorder, since several individuals diagnosed with SCZ will vary in most of the features that describe the disorder (1). Some of them present similar patterns of functional connectivity, which may be related to a similar manifestation of SCZ. Other patients are very different in terms of functional connectivity, possibly due to different manifestations of the psychiatric disorder. The DSM-5 authors have already recognized SCZ as a spectrum of psychiatric disorders, but the diagnostic criteria are still too broad. Subgroups seem to still be present in patients that are diagnosed with SCZ. The same is true for BD and ADHD patients. The three psychiatric disorders included in this study share several characteristic signs and symptoms and seem to share some functional connectivity abnormalities. Therefore, misdiagnosis can be expected.

Nonetheless, this hypothesis is also true for healthy subjects, since the functional connectivity pattern is also not the same for all individuals. Considering that personality traits may be related to patterns of functional connectivity and that those patterns are also dependent on sex, those results are expected as well (50). Even a group of healthy subjects can be split into several subgroups based on personality traits, age, and sex, among other factors. In future studies, it is worth considering the use of unsupervised clustering models as a step following normative models. This way, investigating the existence of subgroups within the same psychiatric disorder becomes possible, allowing for a more individual-level analysis of the psychiatric patients as well.

Also, we may be aware of the limitations of the study, which may hurt the results. First, although the parameters of acquisition that were defined led to the best balance between the amount and quality of data, longer scan duration and higher temporal resolutions are both important factors for more reliable results. Secondly, the template that was used for dual regression was not specific to the data used in this study. Nonetheless, it allowed for a comparison with the literature, and since it included more FBNs than several studies using rs-fMRI in the context of psychiatric disorders, it resulted in findings that are novel to the field of functional interconnectivity analysis. However, this study did not explore intranetwork connectivities, which have been found to be abnormal in a number of psychiatric disorders. Incorporating those metrics could have aided in the improvement of the analysis. Thirdly, the sample size was small, which is a limitation. Fourthly, we started with a simple autoencoder that received no information about sex/age. The model led to good results, but improvements to it could lead to better results. Fifth, we should consider that treatment (medication doses), disease severity, and disease course (stage or duration of onset) impact on the current findings, and a control of such factors could improve this study. Finally, considering that fMRI is a dynamic imaging technique and that the pairs of FBNs are not necessarily physically connected in a direct manner, some pairs of connections may not be as stable as when constructed from structural MRI data and potencially limit the obtained.

Overall, it is demonstrated that functional connectivity has the potential to be used to distinguish patients from healthy subjects and that different psychiatric disorders are characterized by different functional connectivity patterns in the context of a normative model. Besides, the results of this study demonstrate that the group-level differences do not resist to the individual-level analysis. Therefore, the concept of group-pattern disguises the interindividual differences presented in the same psychiatric disorder group. This study also supports the hypothesis that the average patient concept falls apart at the individual-level in the field of psychiatry (51, 52). The findings show that to better understand the neuropathology of psychiatric disorders and develop effective therapeutic approaches, researchers must look beyond the average patient. Considering that psychiatric disorders develop over a continuous spectrum, a precision-based approach focused on the specific functional network changes of individual patients would lead to a more effective diagnosis. The DL-based normative model approach used in this study may be considered for that purpose. It establishes a bridge between DL models and precision medicine, which may be the key to the translation of these models to clinical practice. The normative model approach provides an intuitive match between the manifestations of the disorder and the brain abnormalities, which was not explored in this study. It would be feasible to practice more precise, person-centered care by looking beyond the group-level findings and connecting the clinical symptoms of the patients to the functional connectivity abnormalities identified by the normative model. This association could also lead to the finding of several subgroups within each psychiatric disorder.

## Data availability statement

Publicly available datasets were analyzed in this study. This data can be found here: 1000 Functional Connectomes Project: Consortium for Reliability and Reproducibility (https://fcon_1000.projects.nitrc.org/indi/CoRR/html/), The Mind Research Network COBRE http://fcon_1000.projects.nitrc.org/indi/retro/cobre.html, and from the OpenNeuro platform: UCLA Consortium for Neuropsychiatric Phenomics LA5c Study https://openneuro.org/datasets/ds000030/versions/00016.

## Author contributions

DO-S and HAF contributed to the conception and design of the study. DO-S organized the database, performed the statistical analysis, and wrote the first draft of the manuscript. Both authors contributed to manuscript revision, read, and approved the submitted version.

## References

[1] American Psychiatric Association. Diagnostic and Statistical Manual of Mental Disorders 5. 5th edition. Washington, DC: American Psychiatric Association (2013).

[2] García-Gutiérrez MS Navarrete F Sala F Gasparyan A Austrich-Olivares A Manzanares J. Biomarkers in psychiatry: concept, definition, types and relevance to the clinical reality. Front Psychiatry. (2020) 11:432. 10.3389/fpsyt.2020.00432 32499729 PMC7243207

[3] Vermani M Marcus M Katzman MA. Rates of detection of mood and anxiety disorders in primary care: a descriptive, cross-sectional study. Prim Care Companion CNS Disord. (2011) 13:e1–e10. 10.4088/PCC.10m01013 21977354 PMC3184591

[4] Ayano G Demelash S Yohannes Z Haile K Tulu M Assefa D. Misdiagnosis, detection rate, and associated factors of severe psychiatric disorders in specialized psychiatry centers in Ethiopia. Ann Gen Psychiatry. (2021) 20:10. 10.1186/s12991-021-00333-7 33531016 PMC7856725

[5] Abramovitch A. Misdiagnosis of ADHD in Individuals diagnosed with obsessive-compulsive disorder: guidelines for practitioners. Curr Treat Opt Psychiatry. (2016) 3:225–34. 10.1007/s40501-016-0084-7

[6] Vieira S Pinaya W Mechelli A. Using deep learning to investigate the neuroimaging correlates of psychiatric and neurological disorders: methods and applications. Neurosci Biobehav Rev. (2017) 74:58–75. 10.1016/j.neubiorev.2017.01.002 28087243

[7] Keshavan MS Morris DW Sweeney JA Pearlson G Thaker G Seidman LJ. A dimensional approach to the psychosis spectrum between bipolar disorder and shizophrenia: the schizo-bipolar scale. Schizophr Res. (2011) 133:250–4. 10.1016/j.schres.2011.09.005 21996268 PMC3381911

[8] Pinaya W Mechelli A Sato JR. Using deep autoencoders to identify abnormal brain structural patterns in neuropsychiatric disorders: a large-scale multi-sample study. Hum Brain Mapp. (2019) 40:944–54. 10.1002/hbm.24423 30311316 PMC6492107

[9] Pinaya WHL Scarpazza C Garcia-Dias R Vieira S Baecker L F da Costa P. Using normative modelling to detect disease progression in mild cognitive impairment and Alzheimer's disease in a cross-sectional multi-cohort study. Sci Rep. (2021) 11:15746. 10.1038/s41598-021-95098-0 34344910 PMC8333350

[10] Marquand AF Kia SM Zabihi M Wolfers T Buitelaar JK Beckmann CF. Conceptualizing mental disorders as deviations from normative functioning. Mol Psychiatry. (2019) 24:1415–24. 10.1038/s41380-019-0441-1 31201374 PMC6756106

[11] Kazeminia S Baur C Kuijper A van Ginneken B Navab N Albarqouni S. GANs for medical image analysis. Art Intell Med. (2020) 109:101938. 10.1016/j.artmed.2020.101938 34756215

[12] Kia SM Marquand AF. Neural processes mixed–effect models for deep normative modeling of clinical neuroimaging data. In: Proceedings of the 2nd International Conference on Medical Imaging with Deep Learning. Vol. 102. London: PMLR (2019). p. 297–314.

[13] Marquand AF Rezek I Buitelaar J Beckmann CF. Understanding heterogeneity in clinical cohorts using normative models: beyond case-control studies. Biol Psychiatry. (2016) 80:552–61. 10.1016/j.biopsych.2015.12.023 26927419 PMC5023321

[14] Kessler D Angstadt M Sripada C. Growth charting of brain connectivity networks and the identification of attention impairment in youth. JAMA Psychiatry. (2016) 73:481–9. 10.1001/jamapsychiatry.2016.0088 27076193 PMC5507181

[15] Song P Zha M Yang Q Zhang Y Li X Rudan I. The prevalence of adult attention-deficit hyperactivity disorder: a global systematic review and meta-analysis. J Glob Health. (2021) 11:04009. 10.7189/jogh.11.04009 33692893 PMC7916320

[16] Castellanos FX Aoki Y. Intrinsic functional connectivity in attention-deficit/hyperactivity disorder: a science in development. Biol Psychiatry Cogn Neurosci Neuroimaging. (2016) 1:253–61. 10.1016/j.bpsc.2016.03.004 27713929 PMC5047296

[17] Castellanos FX Margulies DS Kelly C Uddin LQ Ghaffari M Kirsch A. Cingulate - precuneus interactions: a new locus of dysfunction in adult attention-deficit/hyperactivity disorder. Biol Psychiatry. (2008) 63:332–7. 10.1016/j.biopsych.2007.06.025 17888409 PMC2745053

[18] Sripada C Kessler D Fang Y Welsh RC Prem Kumar K Angstadt M. Disrupted network architecture of the resting brain in attention-deficit/hyperactivity disorder. Hum Brain Mapp. (2014) 35:4693–705. 10.1002/hbm.22504 24668728 PMC6869736

[19] Kessler D Angstadt M Welsh RC Ipada C. Modality-spanning deficits in attention-deficit/hyperactivity disorder in functional networks, gray matter, and white matter. J Neurosci. (2014) 34:16555–66. 10.1523/JNEUROSCI.3156-14.2014 25505309 PMC4261086

[20] Sun L Cao Q Long X Sui M Cao X Zhu C. Abnormal functional connectivity between the anterior cingulate and the default mode network in drug-naïve boys with attention deficit hyperactivity disorder. Psychiatry Res. (2012) 201:120–7. 10.1016/j.pscychresns.2011.07.001 22424873

[21] Sutcubasi B Metin B Kurban MK Metin ZE Beser B Sonuga-Barke E. Resting-state network dysconnectivity in ADHD: a system-neuroscience-based meta-analysis. World J Biol Psychiatry. (2020) 21:662–72. 10.1080/15622975.2020.1775889 32468880

[22] Aboitiz F Ossandón T Zamorano F Palma B Carrasco X. Irrelevant stimulus processing in ADHD: catecholamine dynamics and attentional networks. Front Psychol. (2014) 5:183. 10.3389/fpsyg.2014.00183 24723897 PMC3972460

[23] Lin H Lin Q Li H Wang M Chen H Liang Y. Functional connectivity of attention-related networks in drug-naive children with ADHD. J Atten Disord. (2021) 25:377–88. 10.1177/1087054718802017 30259777

[24] Brites C. ADHD and impact on language. In:Kumperščak HG, editor. ADHD–From Etiology to Comorbidity. London: IntechOpen (2020). 10.5772/intechopen.93541

[25] Yoon S Kim TD Kim J Lyoo IK. Altered functional activity in bipolar disorder: a comprehensive review from a large-scale network perspective. Brain Behav. (2021) 11:e01953. 10.1002/brb3.1953 33210461 PMC7821558

[26] Wang J Wang Y Huang H Jia Y Zheng S Zhong S. Abnormal intrinsic brain functional network dynamics in unmedicated depressed bipolar II disorder. J Affect Disord. (2019) 253:402–9. 10.1016/j.jad.2019.04.103 31103805

[27] Anand A Li Y Wang Y Lowe MJ Dzemidzic M. Resting state corticolimbic connectivity abnormalities in unmedicated bipolar disorder and unipolar depression. Psychiatry Res. (2009) 171:189–98. 10.1016/j.pscychresns.2008.03.012 19230623 PMC3001251

[28] Skåtun KC Kaufmann T Brandt CL Doan NT Alnæs D Tønnesen S. Thalamo-cortical functional connectivity in schizophrenia and bipolar disorder. Brain Imaging Behav. (2018) 12:640–52. 10.1007/s11682-017-9714-y 28444556

[29] Lois G Linke J Wessa M. Altered functional connectivity between emotional and cognitive resting state networks in euthymic bipolar I disorder patients. PLoS ONE. (2014) 9:e107829. 10.1371/journal.pone.0107829 25343370 PMC4208743

[30] Friston K Frith C. Schizophrenia: a disconnection syndrome? Clini Neurosci. (1995) 3:89–97. 7583624

[31] Garrity AG Pearlson GD McKiernan K Lloyd D Kiehl KA Calhoun VD. Aberrant “default mode” functional connectivity in schizophrenia. Am J Psychiatry. (2007) 164:450–7. 10.1176/ajp.2007.164.3.450 17329470

[32] Wang Y Tang W Fan X Zhang J Geng D Jiang K. Resting-state functional connectivity changes within the default mode network and the salience network after antipsychotic treatment in early-phase schizophrenia. Neuropsychiatr Dis Treat. (2017) 13:397–406. 10.2147/NDT.S123598 28223812 PMC5308583

[33] Liang M Zhou Y Jiang T Liu Z Tian L Liu H. Widespread functional disconnectivity in schizophrenia with resting-state functional magnetic resonance imaging. NeuroReport. (2006) 17:209–13. 10.1097/01.wnr.0000198434.06518.b8 16407773

[34] Bernard JA Russell CE Newberry RE Goen JRM Mittal VA. Patients with schizophrenia show aberrant patterns of basal ganglia activation: evidence from ALE meta-analysis. NeuroImage Clin. (2017) 14:450–63. 10.1016/j.nicl.2017.01.034 28275545 PMC5328905

[35] Li P Fan TT Zhao RJ Han Y Shi L Sun HQ. Altered brain network connectivity as a potential endophenotype of schizophrenia. Sci Rep. (2017) 7:5483. 10.1038/s41598-017-05774-3 28710394 PMC5511161

[36] Zuo XN Anderson JS Bellec P Birn RM Biswal BB Blautzik J. An open science resource for establishing reliability and reproducibility in functional connectomics. Sci Data. (2014) 1:140049. 10.1038/sdata.2014.49 25977800 PMC4421932

[37] Gorgolewski KJ Durnez J Poldrack RA. Preprocessed consortium for neuropsychiatric phenomics dataset. F1000Research. (2017) 6:1262. 10.12688/f1000research.11964.2 29152222 PMC5664981

[38] Mayer AR Ruhl D Merideth F Ling J Hanlon FM Bustillo J. Functional imaging of the hemodynamic sensory gating response in schizophrenia. Hum Brain Mapp. (2013) 34:2302–12. 10.1002/hbm.22065 22461278 PMC4020570

[39] Stephen JM Coffman BA Jung RE Bustillo JR Aine CJ Calhoun VD. Using joint ICA to link function and structure using MEG and DTI in schizophrenia. NeuroImage. (2013) 83:418–30. 10.1016/j.neuroimage.2013.06.038 23777757 PMC3815989

[40] Hanlon FM Houck JM Pyeatt CJ Lundy SL Euler MJ Weisend MP. Bilateral hippocampal dysfunction in schizophrenia. NeuroImage. (2011) 58:1158–68. 10.1016/j.neuroimage.2011.06.091 21763438 PMC4063352

[41] Calhoun VD Sui J Kiehl K Turner J Allen E Pearlson G. Exploring the psychosis functional connectome: aberrant intrinsic networks in schizophrenia and bipolar disorder. Front Psychiatry. (2012) 2:75. 10.3389/fpsyt.2011.00075 22291663 PMC3254121

[42] Avants B Tustison N Song G. Advanced normalization tools: V1.0. Insight J. (2009). 10.54294/uvnhin

[43] Jenkinson M Beckmann CF Behrens TEJ Woolrich MW Smith SM. FSL. NeuroImage. (2012) 62:782–90. 10.1016/j.neuroimage.2011.09.015 21979382

[44] Beckmann CF Smith SM. Probabilistic independent component analysis for functional magnetic resonance imaging. IEEE Trans Med Imaging. (2004) 23:137–52. 10.1109/TMI.2003.822821 14964560

[45] Bijsterbosch J Smith S Beckmann C. Introduction to Resting State fMRI Functional Connectivity. 1st edition. Oxford: Oxford Neuroimaging Primers (2017).

[46] Wylie GR Genova H Deluca J Chiaravalloti N Sumowski JF. Functional magnetic resonance imaging movers and shakers: does subject-movement cause sampling bias? Hum Brain Mapp. (2014) 35:1–13. 10.1002/hbm.22150 22847906 PMC3881195

[47] Xiong H Guo RJ Shi HW. Altered default mode network and salience network functional connectivity in patients with generalized anxiety disorders: an ICA-based resting-state fMRI study. Evid Based Complement Alternat Med. (2020) 2020:4048916. 10.1155/2020/4048916 32855650 PMC7443230

[48] Shirer WR Ryali S Rykhlevskaia E Menon V Greicius MD. Decoding subject-driven cognitive states with whole-brain connectivity patterns. Cereb Cortex. (2012) 22:158–65. 10.1093/cercor/bhr099 21616982 PMC3236795

[49] Thomas Yeo BT Krienen FM Sepulcre J Sabuncu MR Lashkari D Hollinshead M. The organization of the human cerebral cortex estimated by intrinsic functional connectivity. J Neurophysiol. (2011) 106:1125–65. 10.1152/jn.00338.2011 21653723 PMC3174820

[50] Nostro AD Müller VI Varikuti DP Pläschke RN Hoffstaedter F Langner R. Predicting personality from network-based resting-state functional connectivity. Brain Struct Funct. (2018) 223:2699–719. 10.1007/s00429-018-1651-z 29572625 PMC5997535

[51] Wolfers T Doan NT Kaufmann T Alnæs D Moberget T Agartz I. Mapping the heterogeneous phenotype of schizophrenia and bipolar disorder using normative models. JAMA Psychiatry. (2018) 75:1146–55. 10.1001/jamapsychiatry.2018.2467 30304337 PMC6248110

[52] Zabihi M Oldehinkel M Wolfers T Frouin V Goyard D Loth E. Dissecting the heterogeneous cortical anatomy of autism spectrum disorder using normative models. Biol Psychiatry Cogn Neurosci Neuroimaging. (2019) 4:567–78. 10.1016/j.bpsc.2018.11.013 30799285 PMC6551348

[53] Oliveira-Saraiva D. Normative model for the diagnosis of neuropsychiatric disorders using deep learning methods. (Master's thesis). Lisboa, Portugal: University of Lisbon (2021).

