# Supplementary material for: Normative model detects abnormal functional connectivity in psychiatric disorders
Source: Front Psychiatry. 2023 Feb 15;14:1068397. doi: 10.3389/fpsyt.2023.1068397 (PMC9975396; doi:10.3389/fpsyt.2023.1068397)
Supplement: Supplementary file 1 [file Data_Sheet_1.pdf]

frontiers

Supplementary Material

Correspondence*:

1 SUPPLEMENTARY DATA

1.1 Additional information about the Shirer *et al* (1) template

In the context of this study, the fourteen functional brain networks (FBNs) that were identified in the study by Shirer *et al* (1) were used as a template, and are the main reference in terms of nomenclature, location, and function.

Nonetheless, the study by Yeo *et al* (2) needs to be referred, as the seven FBNs that they identified are widely cited in the literature. Thus, it is important to match the FBNs from both studies to better understand the agreement between the results of this study and previous findings in the literature. This step was performed using FSLeyes, and the results are presented in Figures 1 and 2.

The anterior salience network (ASN) from Shirer *et al* (1) matches with parts of the ventral attention network (VAN) from Yeo *et al* (2). The auditory network (AN) from Shirer *et al* (1) matches with parts of the somatomotor network (SON) from Yeo *et al* (2). The dorsal default mode network (DDMN) and the language network (LN) from Shirer *et al* (1) match with parts of the default mode network (DMN) from Yeo *et al* (2). The higher visual network (HVN) and primary visual network (PVN) from Shirer *et al* (1) match with parts of the visual network (VIN) from Yeo *et al* (2). The left executive control network (LECN) and the right executive control network (RECN) from Shirer *et al* (1) match with the frontoparietal network (FPN) from Yeo *et al* (2), and also have some regions that overlap with the DMN from Yeo *et al* (2). The posterior salience network (PSN) from Shirer *et al* (1) matches with a few parts of the VAN, and may also be represented by parts of the FPN from Yeo *et al* (2). The precuneus network (PN) from Shirer *et al* (1) matches with both the DMN and FPN from Yeo *et al* (2). The sensorimotor network (SN) from Shirer *et al* (1) matches with parts of the somatomotor network (SON) from Yeo *et al* (2), and also have some regions that overlap with the limbic network (LIM) from Yeo *et al* (2). The ventral default mode network (VDMN) from Shirer *et al* (1) matches with both the DMN and the dorsal attention network (DAN) from Yeo *et al* (2). The visuospatial network (VN) from Shirer *et al* (1) matches with several parts of the DAN from Yeo *et al* (2). The basal ganglia network (BGN) from Shirer *et al* (1) is not present in the study from Yeo *et al* (2).

Afterward, it is possible to associate those FBNs with their anatomy and function.

The ASN encompasses the dorsal anterior cingulate cortex and the anterior insula. These regions of the FBN are activated in the presence of several heterogeneous stimuli, and this FBN plays a role in the linking between cognition and emotion/interoception processes (3). The dorsal anterior cingulate cortex structure is associated with executive control, learning, adjustment, economic choice, and self-control (4). The anterior insula mediates interoceptive attention (5).

The AN is located in the primary auditory cortex and is related to audition, hearing music and speech processes (3).

The BGN includes the basal ganglia and thalamus, and it is linked with motor, pain, and somatosensory processes, and a wide range of mental processes, including reward tasks, classical conditioning, and anxiety

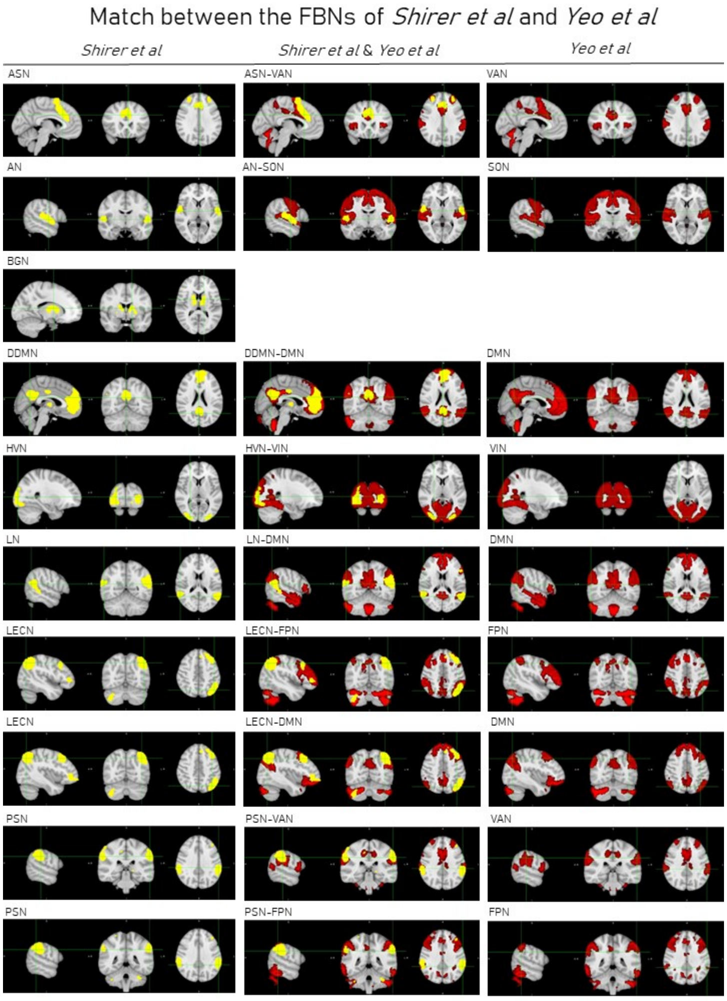

Figure 1. Match between the FBNs of Shirer *et al* (1) and Yeo *et al* (2) (I). FBNs from Shirer *et al* (1): ASN- anterior salience network; AN- auditory network; BGN- basal ganglia network; DDMN- dorsal default mode network; HVN- higher visual network; LN- language network; LECN- left executive control network; PSN- posterior salience network; PN- precuneus network; PVN- primary visual network; RECN- right executive control network; SN- sensorimotor network; VDMN- ventral default mode network; VN- visuospatial network. Additionally information about the anatomy of FBNs can be found in Figure 3. FBNs from Yeo *et al* (2): DMN- default mode network; DAN- dorsal attention network; FPN- frontoparietal network; LIM- limbic network; SON- somatomotor network; VAN- ventral attention network; VIN- visual network.

(3). The basal ganglia refer to the caudate, putamen, globus pallidus, nucleus accumbens, substantia nigra and subthalamic nucleus. These structures are responsible for controlling voluntary movements, cognition, and emotional processes. The thalamus acts as a relay station that receives, processes, and sends sensory information to an associated cortical area (6).

The DDMN corresponds to the posterior cingulate cortex, medial prefrontal cortex, angular gyrus, and hippocampus. All regions of this FBN overlap the well-known, DMN, which is possibly the most widely studied FBN with rs-fMRI since it is activated when no task is being done. This FBN is related to internal mental-state processes, such as self-referential processing, interoception, autobiographical memory retrieval, or imagining the future. (7, 8). The VDMN is located in the medial temporal lobe, retrosplenial cortex, posterior cingulate cortex, and regions of the cerebellum. Several regions of this FBN are also part

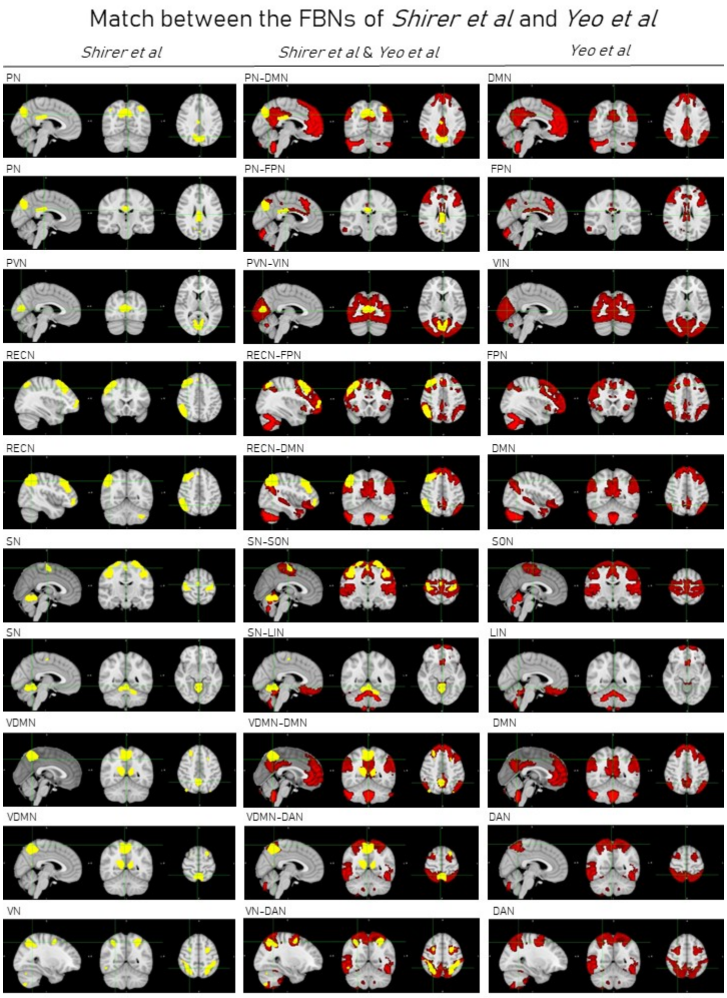

Figure 2. Match between the FBNs of Shirer *et al* (1) and Yeo *et al* (2) (II). FBNs from Shirer *et al* (1): ASN- anterior salience network; AN- auditory network; BGN- basal ganglia network; DDMN- dorsal default mode network; HVN- higher visual network; LN- language network; LECN- left executive control network; PSN- posterior salience network; PN- precuneus network; PVN- primary visual network; RECN- right executive control network; SN- sensorimotor network; VDMN- ventral default mode network; VN- visuospatial network. Additionally information about the anatomy of FBNs can be found in Figure 3. FBNs from Yeo *et al* (2): DMN- default mode network; DAN- dorsal attention network; FPN- frontoparietal network; LIM- limbic network; SON- somatomotor network; VAN- ventral attention network; VIN- visual network

of the DMN. Regarding function, it is interesting to notice that while the VDMN is related to the vividness but not the valence of imagined events, the DDMN is related to the valence but not the vividness of those imagined events (9).

The HVN is located in the secondary visual cortex, which includes the left and right occipital gyri, and is related to higher-level visual processing, such as reading tasks. The PVN is located in the primary visual cortex, at the calcarine sulcus, and is related to simple visual stimuli, such as flashlights (10).

The LN encompasses the inferior frontal gyrus (Broca's area), left middle temporal gyrus, angular gyrus, superior temporal gyrus, and supramarginal gyrus (Wernicke's Area), and is associated with speech and

	Anatomical Location of Functional ROIs
Auditory	Left Superior Temporal Gyrus, Heschl's Gyrus
	Right Superior Temporal Gyrus
	Right Thalamus
Basal Ganglia	Left Thalamus, Caudate
	Right Thalamus, Caudate, Putamen
	Left Inferior Frontal Gyrus
	Right Inferior Frontal Gyrus
	Pons
PCC/MPFC	Medial Prefrontal Cortex, Anterior Cingulate Cortex, Orbitofrontal Cortex
	Left Angular Gyrus
	Right Superior Frontal Gyrus
	Posterior Cingulate Cortex, Precuneus
	Midcingulate Cortex
	Right Angular Gyrus
	Left and Right Thalamus
	Left Hippocampus
	Right Hippocampus
V2	Left Middle Occipital Gyrus, Superior Occipital Gyrus
	Right Middle Occipital Gyrus, Superior Occipital Gyrus
Language	Inferior Frontal Gyrus
	Left Middle Temporal Gyrus
	Left Middle Temporal Gyrus, Angular Gyrus
	Left Middle Temporal Gyrus, Superior Temporal Gyrus, Supramarginal Gyrus, Angular Gyrus
	Right Inferior Frontal Gyrus
	Right Supramarginal Gyrus, Superior Temporal Gyrus, Middle Temporal Gyrus
	Left Crus I
Left DLPFC/Parietal	Left Middle Frontal Gyrus, Superior Frontal Gyrus
	Left Inferior Frontal Gyrus, Orbitofrontal Gyrus
	Left Superior Parietal Gyrus, Inferior Parietal Gyrus, Precuneus, Angular Gyrus
	Left Inferior Temporal Gyrus, Middle Temporal Gyrus
	Right Crus I
	Left Thalamus
Sensorimotor	Left Precentral Gyrus, Postcentral Gyrus
	Right Precentral Gyrus, Postcentral Gyrus
	Right Supplementary Motor Area
	Left Thalamus
	Bilateral Lobule IV, Lobule V, Lobule VI
	Right Thalamus
Posterior Insula	Left Middle Frontal Gyrus
	Left Supramarginal Gyrus, Inferior Parietal Gyrus
	Left Precuneus
	Right Midcingulate Cortex
	Right Superior Parietal Gyrus, Precuneus
	Right Supramarginal Gyrus, Inferior Parietal Gyrus
	Left Thalamus
	Lobule VI
	Left Posterior Insula, Putamen
	Right Thalamus
	Lobule VI
	Right Posterior Insula
Precuneus	Midcingulate Cortex, Posterior Cingulate Cortex
	Precuneus
	Left Angular Gyrus
	Right Angular Gyrus
V1	Calcarine Sulcus
	Left Thalamus
Right DLPFC/Parietal	Right Middle Frontal Gyrus, Right Superior Frontal Gyrus
	Right Middle Frontal Gyrus
	Right Inferior Parietal Gyrus, Supramarginal Gyrus, Angular Gyrus
	Right Superior Frontal Gyrus
	Left Crus I, Crus II, Lobule VI
	Right Caudate
Insula/dACC	Left Middle Frontal Gyrus
	Left Insula
	Anterior Cingulate Cortex, Medial Prefrontal Cortex, Supplementary Motor Area
	Right Middle Frontal Gyrus
	Right Insula
	Left Lobule VI, Crus I
	Right Lobule VI, Crus I
RSC/MTL	Left Retrosplenial Cortex, Posterior Cingulate Cortex
	Left Middle Frontal Gyrus
	Left Parahippocampal Gyrus
	Left Middle Occipital Gyrus
	Right Retrosplenial Cortex, Posterior Cingulate Cortex
	Precuneus
	Right Superior Frontal Gyrus, Middle Frontal Gyrus
	Right Parahippocampal Gyrus
	Right Angular Gyrus, Middle Occipital Gyrus
	Right Lobule IX
IPS/FEF	Left Middle Frontal Gyrus, Superior Frontal Gyrus, Precentral Gyrus
	Left Inferior Parietal Sulcus
	Left Frontal Operculum, Inferior Frontal Gyrus
	Left Inferior Temporal Gyrus
	Right Middle Frontal Gyrus
	Right Inferior Parietal Lobule
	Right Frontal Operculum, Inferior Frontal Gyrus
	Right Middle Temporal Gyrus
	Left Lobule VIII, Lobule VIIb
	Right Lobule VIII, Lobule VIIb
	Right Lobule VI, Crus I

Figure 3. Information about the anatomy of the FBNs from Shirer *et al* (1). Auditory- AN; Basal Ganglia-BGN; Posterior Cingulate Cortex (PCC)/Medial Prefrontal Cortex (MPFC)- DDMN; Secondary Visual Cortex (V2)- HVN; Language- LN; Left Dorsolateral Prefrontal Cortex (DLPFC)/Left Parietal Lobe - LECN; Sensorimotor- SN; Posterior Insula- PSN; Precuneus- PN; Primary Visual Cortex (V1)- PVN; Right Dorsolateral Prefrontal Cortex (DLPFC)/Right Parietal Lobe- RECN; Insula/Dorsal Anterior Cingulate Cortex (dACC)- ASN; Retrosplenial Cortex (RSC)/Medial Temporal Lobe (MTL)- VDMN; Intraparietal Sulcus (IPS)/Frontal Eye Field (FEF) - VN (Figure reproduced with permission from Shirer *et al* (1))

language processes. While Broca's area is mostly associated with the production of language, Wernicke's area is mostly related to the understanding of spoken language (10).

The LECN and RECN are located in left/right frontoparietal regions, including the left/right dorsolateral prefrontal cortex, posterior parietal, and cerebellum regions, respectively. Those FBNs are essential for coordinating behavior efficiently (11). They are related to several cognition paradigms that require externally directed attention, such as working memory, relational integration, response inhibition, and task-set switching. Regions of those FBNs have also been related to several creative thought processes. While the RECN appears to be involved in multiple cognitive processes, such as reasoning, attention, inhibition, and memory, the LECN is strongly related to working memory, and language tasks (12, 3).

The PSN is mostly located in the posterior insula. This region represents interoceptive information about the body's physiological status and appears to play a role in the recognition, intensity encoding, localization, learning, and memory of painful events (13).

Although the PN is mostly located in the precuneus, it also involves the midcingulate cortex and posterior cingulate cortex. The precuneus is involved in complex functions, such as integration of information relating to environmental perception, mental imagery strategies, episodic memory retrieval, affective responses to pain, and sense of self (14, 15).

The SN includes somatosensory (postcentral gyrus) and motor (precentral gyrus) regions and extends to the supplementary motor area, and is related to motor tasks (16).

The VN is located in the intraparietal sulcus and frontal eye fields and is activated when attention is oriented in space, such as when planning eye movements, grasping movements, or defensive head movements (17, 18).

Further information about the anatomy of those FBNs can be found in Figure 3.

1.2 Analysis of the pairs of FBNs that characterize the patients' sets for smaller and larger H-Test-U sets

The H-Test-U set was used in this study to test the ability of the algorithm to learn specific healthy characteristics and, consequently, the ability to characterize the different patient groups. Considering that, further analysis with a larger H-Test-U set would enable us to obtain more robust results regarding the pairs of FBNs that characterize each group of patients. This analysis involved comparing the results from a smaller and a larger (double the size) H-Test-U sets. Therefore, two training/test sizes were considered: a) the original model with a H-Test-U set with 39 healthy individuals and a H-Train set with 366 healthy individuals; b) the model with a H-Test-U set with 78 healthy individuals and a H-Train set with 327 healthy individuals.

As such, here Figure 4 shows a comparison of the matrices representing the MSE_f for each pair of FBNs, considering all the four UCLA test sets for the two training/test sizes considered.

Figure 5 shows the comparison of the matrices representing the MSE_f of the SCZ-Test-U, BD-Test-U, and ADHD-Test-U matrices of Figure 4 subtracted by the MSE_f H-Test-U matrix, for the two training/test sizes considered.

Finally, Figure 6 displays the comparison of the connectograms showing the 10% of pairs of FBNs that were worse reconstructed for each group without and with subtraction of the H-Test-U MSEf matrix, for the two training/test sizes considered.

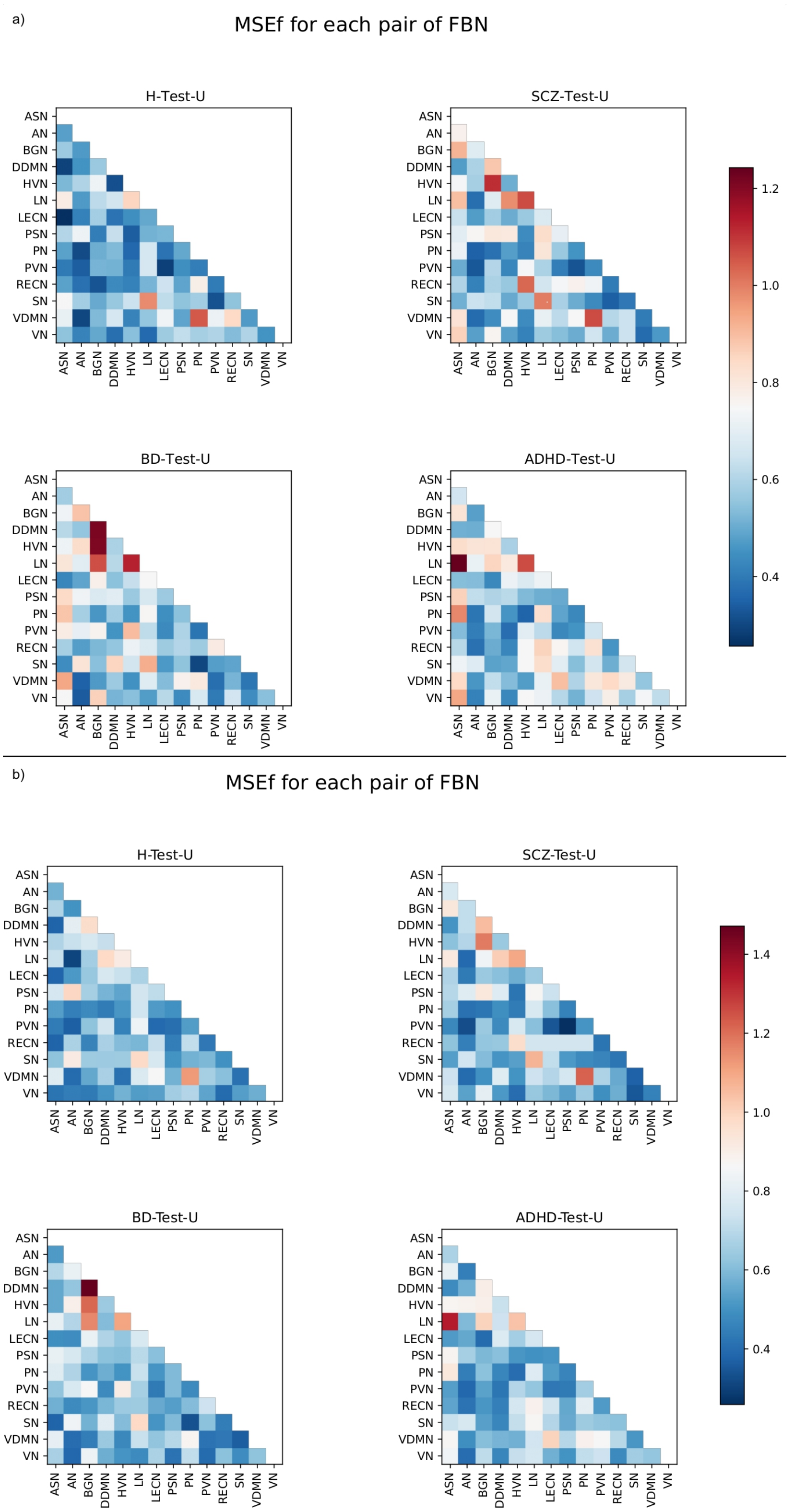

Figure 4. Comparison between the matrices representing the MSE_f for each pair of FBNs, for the four test sets of the UCLA dataset, considering: a) H-Test-U and H-Train with 39 and 366 healthy individuals, respectively; b) H-Test-U and H-Train with 78 and 327 healthy individuals, respectively. For each analysis, the color bars range between the lowest and the highest values of the MSEf for each pair of FBNs of all test sets.

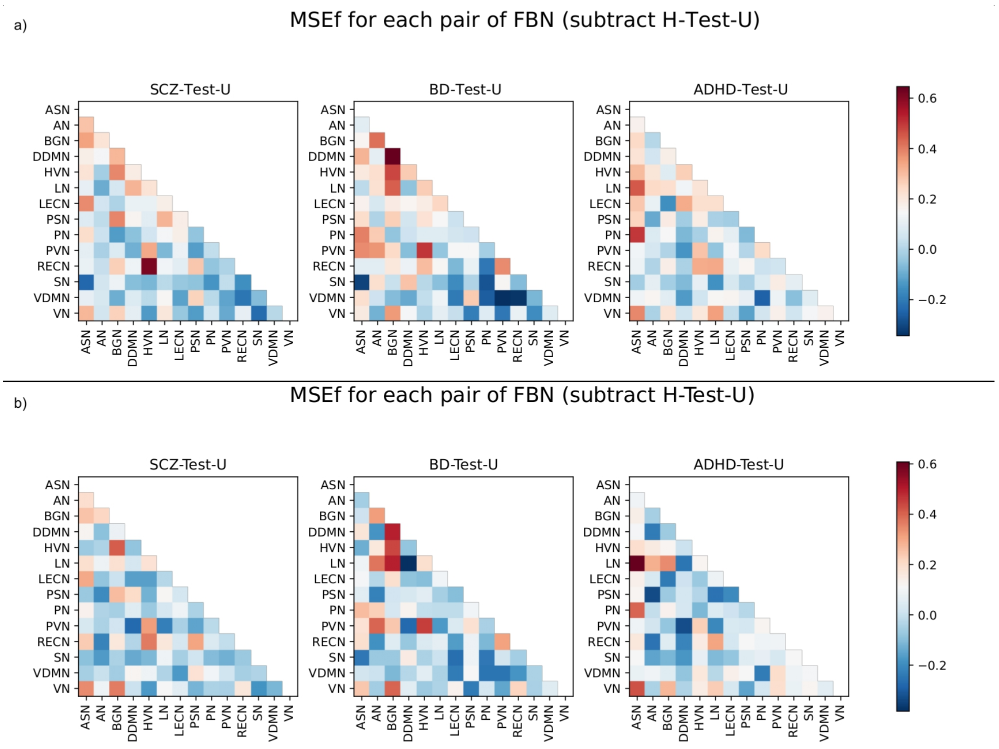

Figure 5. Comparison between the matrices representing the MSE_f SCZ-Test-U, BD-Test-U, and ADHD-Test-U matrices represented in Figure 4, subtracted by the H-Test-U matrix, considering: a) H-Test-U and H-Train with 39 and 366 healthy individuals, respectively; b) H-Test-U and H-Train with 78 and 327 healthy individuals, respectively. For each analysis, the color bars range between the lowest and the highest values of the MSE_f for each pair of FBNs of all test sets after subtraction of the H-Test-U MSE_f matrix.

Overall, the results were similar for both cases. The MSE_f values were higher for the model with a larger H-Test-U set, as would be expected. This is justified by the reduction of the size of the training set, which made it harder for the model to reconstruct outputs that were similar to the inputs.

Table 1 shows that the majority of the 10% of FBNs pairs that were worse reconstructed considering the smaller and larger H-Test-U sets were the same for each patient group. The remaining FBNs in the 10% selection, have nonetheless high MSEf for both cases and for each patient group, suggesting that disease-characteristic FBNs are considerably robust, independently of the size of the healthy training and test sets.

For subsequent analysis, only those 10% of FBNs commonly worse reconstructed were considered to provide higher confidence.

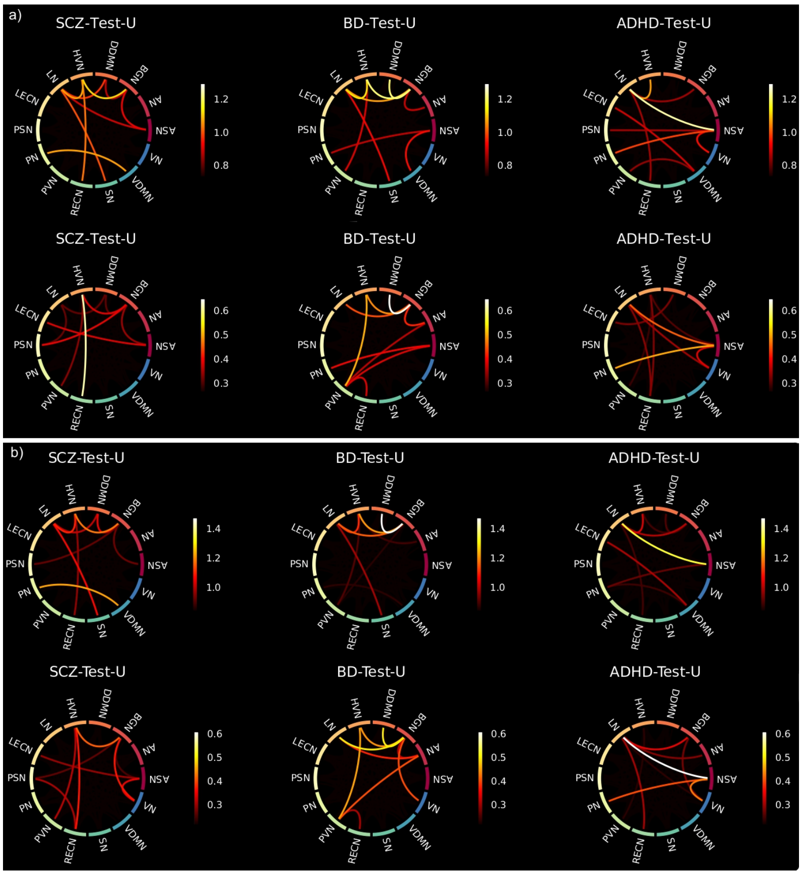

Figure 6. Comparison between the connectograms showing the 10% of pairs of FBNs that were worse reconstructed for each group without (top) and with (bottom) subtraction of the H-Test-U MSEf matrix, considering: a) H-Test-U and H-Train with 39 and 366 healthy individuals, respectively; b) H-Test-U and H-Train with 78 and 327 healthy individuals, respectively. For each analysis, the color bars range between the lowest and highest values of those 10% of pairs of FBNs extracted from Figures 4 and 5, respectively.

Table 1. Analysis of the pairs of FBNs that were and were not kept for the MSE_f connectograms of each patient set (SCZ-Test-U, BD-Test-U, ADHD-Test-U), and for those patient sets subtracted by the MSE_f H-Test-U matrix (SCZ-Test-U - H-Test-U, BD-Test-U - H-Test-U, ADHD-Test-U - H-Test-U), considering: a) H-Test-U and H-Train with 39 and 366 healthy individuals, respectively; b) H-Test-U and H-Train with 78 and 327 healthy individuals, respectively.

MSEf Connectogram	Pairs of FBNs kept for both H-Test-U sizes	Pairs of FBNs only present for the H-Test-U (39 subjects)	Pairs of FBNs only present for the H-Test-U (78 subjects)
**SCZ-Test-U**	ASN-BGN, BGN-DDMN, BGN-HVN, DDMN-LN, HVN-LN, HVN-RECN, LN-SN, PN-VDMN	ASN-LN	BGN-PSN
**SCZ-Test-U - H-Test-U**	ASN-BGN, BGN-HVN, ASN-LECN, BGN-PSN, HVN-PVN, HVN-RECN	DDMN-LN, LN-PSN, BGN-DDMN	ASN-VN, PSN-RECN, BGN-VN
**BD-Test-U**	BGN-DDMN, BGN-HVN, BGN-LN, HVN-LN, HVN-PVN, LN-PSN	ASN-PN, ASN-VDMN, AN-BGN	AN-HVN, BGN-DDMN, PN-VDMN
**BD-Test-U - H-Test-U**	AN-BGN, BGN-DDMN, BGN-HVN, BGN-LN, AN-PVN, HVN-PVN, RECN-PVN	ASN-PVN, ASN-PN	AN-LN, BGN-VN
**ADHD-Test-U**	ASN-LN, BGN-LN, HVN-LN, ASN-PN, LN-RECN, LECN-VDMN	ASN-PSN, PVN-VDMN, ASN-VN	BGN-DDMN, BGN-HVN, PN-VDMN
**ADHD-Test-U - H-Test-U**	ASN-LN, ASN-PN, HVN-PVN, LN-RECN, ASN-VN, LN-VN	DDMN-LECN, HVN-RECN, ASN-HVN	BGN-LN, BGN-VN, AN-LN

REFERENCES

1 .Shirer WR, Ryali S, Rykhlevskaia E, Menon V, Greicius MD. Decoding subject-driven cognitive states with whole-brain connectivity patterns. *Cerebral Cortex* **22** (2012) 158–165. doi:10.1093/cercor/bhr099.

2 .Thomas Yeo BT, Krienen FM, Sepulcre J, Sabuncu MR, Lashkari D, Hollinshead M, et al. The organization of the human cerebral cortex estimated by intrinsic functional connectivity. *Journal of Neurophysiology* **106** (2011) 1125–1165. doi:10.1152/jn.00338.2011.

3 .Laird AR, Fox PM, Eickhoff SB, Turner JA, Ray KL, Mckay DR, et al. Behavioral interpretations of intrinsic connectivity networks. *Journal of Cognitive Neuroscience* **23** (2011) 4022–4037. doi:10.1162/jocn_a_00077.

4 .Voloh B, Knoebl R, Hayden BY, Zimmermann J. Oscillations as a window into neuronal mechanisms underlying dorsal anterior cingulate cortex function. *International Review of Neurobiology* **158** (2021) 311–335. doi:10.1016/bs.irn.2020.11.003.

5 .Wang X, Wu Q, Egan L, Gu X, Liu P, Gu H, et al. Anterior insular cortex plays a critical role in interoceptive attention. *eLife* **8** (2019) e42265. doi:10.7554/ELIFE.42265.

6 .Torrico T, Munakomi S. Neuroanatomy, thalamus. *StatPearls* (StatPearls Publishing) (2021).

7 .Smith SM, Fox PT, Miller KL, Glahn DC, Fox PM, Mackay CE, et al. Correspondence of the brain's functional architecture during activation and rest. *Proceedings of the National Academy of Sciences of the United States of America* **106** (2009) 13040–13045. doi:10.1073/pnas.0905267106.

8 .Ekhtiari H, Nasseri P, Yavari F, Mokri A, Monterosso J. Neuroscience of drug craving for addiction medicine: From circuits to therapies. *Progress in Brain Research* **223** (2016) 115–141. doi:10.1016/bs.pbr.2015.10.002.

9 .Lee S, Parthasarathi T, Kable JW. The ventral and dorsal default mode networks are dissociably modulated by the vividness and valence of imagined events. *Journal of Neuroscience* **41** (2021) 5243–5250. doi:10.1523/JNEUROSCI.1273-20.2021.

10 .Purves D, Augustine GJ, Fitzpatrick D, Hall WC, LaMantia AS, McNamara JO, et al. *Neuroscience* (Massachusetts: Sinauer Associates Inc Publishers), 3rd edn. (2004).

11 .Marek S, Dosenbach NU. The frontoparietal network: function, electrophysiology, and importance of individual precision mapping. *Dialogues in Clinical Neuroscience* **20** (2018) 133. doi:10.31887/DCNS.2018.20.2/smarek.

12 .Beaty RE, Benedek M, Barry Kaufman S, Silvia PJ. Default and executive network coupling supports creative idea production. *Scientific Reports* **5** (2015) 10964. doi:10.1038/srep10964.

13 .Coen SJ, Hobson AR, Aziz Q. Processing of gastrointestinal sensory signals in the brain. *Physiology of the Gastrointestinal Tract* (Academic Press), vol. 1. 5th edn. (2012), 689–702. doi:10.1016/B978-0-12-382026-6.00023-3.

14 .Rolls ET. The neuroscience of emotional disorders. *Handbook of Clinical Neurology* (Elsevier), vol. 183 (2021), 1–26. doi:10.1016/B978-0-12-822290-4.00002-5.

15 .Borsook D, Maleki N, Burstein R. Migraine. *Neurobiology of Brain Disorders: Biological Basis of Neurological and Psychiatric Disorders* (Academic Press) (2015), 693–708. doi:10.1016/B978-0-12-398270-4.00042-2.

16 .Chenji S, Jha S, Lee D, Brown M, Seres P, Mah D, et al. Investigating default mode and sensorimotor network connectivity in amyotrophic lateral sclerosis. *PLoS ONE* **11** (2016) e0157443. doi:10.1371/journal.pone.0157443.

17 .Vossel S, Geng JJ, Fink GR. Dorsal and ventral attention systems: Distinct neural circuits but collaborative roles. *The Neuroscientist* **20** (2014) 150–159. doi:10.1177/1073858413494269.

18 .Mai JK, Paxinos G. *The Human Nervous System* (Elsevier Inc.), 3rd edn. (2012). doi:10.1016/C2009-0-02721-4.
